# Several major herb pairs containing *Coptidis rhizoma*: a review of key traditional uses, constituents and compatibility effects

**DOI:** 10.3389/fphar.2024.1399460

**Published:** 2024-06-25

**Authors:** Shi-Yu Li, Ding-Qiao Xu, Yan-Yan Chen, Rui-Jia Fu, Yu-Ping Tang

**Affiliations:** ^1^ Key Laboratory of Shaanxi Administration of Traditional Chinese Medicine for TCM Compatibility, and Shaanxi Key Laboratory of Chinese Medicine Fundamentals and New Drugs Research, Shaanxi University of Chinese Medicine, Xi’an, Shaanxi Province, China; ^2^ Wuxi Institute of Integrated Chinese and Western Medicine, and Wuxi Cancer Institute, Affiliated Hospital of Jiangnan University, Wuxi, Jiangsu Province, China

**Keywords:** *Coptidis rhizoma*, herb pairs, traditional uses, ethnopharmacology, compatibility

## Abstract

Herb compatibility is the soul of traditional Chinese Medicine prescriptions. *Coptidis rhizoma* (CR) (*Coptis chinensis* Franch., *Coptis deltoidea* C.Y.Cheng et Hsiao, or *Coptis teeta* Wall.; family Ranunculaceae), is a well-known herb. The bitter and cold nature of CR can irritate the spleen and stomach, and certain ingredients in CR may trigger allergic reactions. Herb combinations can help alleviate the side effects caused by CR. Through data analysis and literature research, there are many herbs combined with CR have a high frequency, but only a few are currently used as formulae in clinical practice. The results showed that these six herb pairs are usually widely studied or used as prescriptions in the clinic. This paper describes the six herb pairs from the key traditional uses, changes in bioactive constituents, and compatibility effects, especially with *Euodiae fructus* (family Rutaceae), *Scutellariae radix* (family Lamiaceae), *Magnoliae Officinalis cortex* (family Magnoliaceae), *Glycyrrhizae radix et rhizoma* (family Fabaceae), *Ginseng radix et rhizoma* (family Araliaceae), and *Aucklandiae radix* (family Asteraceae), and found that herbs are more effective when used in combination. Therefore, it is feasible to establish some methods to study herb pairs comprehensively from different perspectives. This paper aims to provide the latest and most comprehensive information on the six herb pairs and summarize the pattern of CR compatibility effects. It aims to attract more attention, and further experimental studies will be conducted to investigate and evaluate the effects of herb pairs containing CR. These data can also provide valuable references for researchers and also provide more possibilities for future applications in clinical practice and new drug development.

## Graphical Abstract

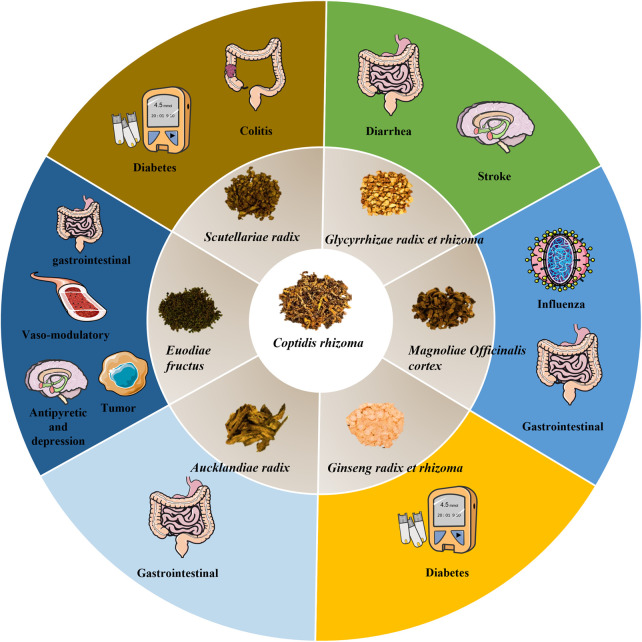

## 1 Introduction

Nowadays, the study of traditional Chinese medicine (TCM) has become a global phenomenon. TCM has a more than 2,000-year history in China and other Eastern nations (Ung et al., 2007). In ancient times, people primarily used single herbs to prevent and treat diseases. As experience has accumulated, doctors have discovered that using single herbs to treat complex diseases has certain limitations. They are also susceptible to side effects or herb resistance mechanisms (Ramsay et al., 2018). The long-term application and clinical experience of physicians have shown that herb compatibility is not only a good choice but also an inevitable one (Li et al., 2010). Herb pair is a commonly used form of herbal compatibility in clinical prescriptions of TCM, which is the long-term summary of medical practice by generations of medical experts (Wang et al., 2012; Tang et al., 2013). It continues to influence modern medicine’s understanding of herb interactions to this day. The compatibility of herbs is essential in TCM prescriptions. Although composed of only two herbs, it cleverly embodies the core idea of traditional Chinese formulas. The compatibility of herbs follows two major principles. Firstly, it involves summarizing and classifying the compatibility of herb pairs based on the theory of the “seven compatibilities,” which focuses on the changes in efficacy before and after the combination of herb pairs. Secondly, it involves understanding the compatibility of herb pairs based on the properties of the herbs themselves and different treatment methods for various diseases (Wang et al., 2013; Tang and Duan, 2014). In short, although an herb pair consists of two herbs, it is not easy to compose. It can withstand the test of clinical practice and has reasonable evidence and principles to follow. In essence, the approach and clinical effects of the herb pairs combination are consistent with the prescription. Therefore, compared to traditional prescriptions, herb pairs not only possess the compatibility characteristics of prescriptions but also offer the advantages of having relatively simple ingredients and being easy to research. *Shennong’s Classic of Materia Medica*, one of the most ancient books in TCM, was the first to summarize the interactions between herbs and identify them as the “seven compatibilities.” They cover almost all possible interactions, including single action, mutual reinforcement, mutual assistance, mutual restraint, mutual detoxication, mutual inhibition, and mutual antagonism. These interactions are based on extensive clinical experience. In these aspects, mutual reinforcement and mutual assistance are the most common forms of compatibility (Jin et al., 2016). These examples fully demonstrate the complex and diverse characteristics of herbs in compatibility. The significance of herb-drug and herb-herb interactions for the safe and efficient use of Chinese traditional medicine has been widely discussed (X. Zhou et al., 2021). More and more people are showing a strong interest in the compatibility of herbs. Analyzing the interactions between various herbs in traditional Chinese formulas through pharmacokinetics, genomics, and other methods not only reveals the pharmacological basis of traditional Chinese formulas but also helps discover new compatibility patterns. Research on herb pairs may serve as the foundation and entry point for comprehensive studies on the compatibility of Chinese herbal medicines (Liang et al., 2011). It can be seen that the study of compatibility characteristics of herb pairs and clinical application rules has significant theoretical significance and practical value for analyzing the composition of prescriptions and mastering drug use laws. This is conducive to the development of new drugs (Duan et al., 2009). Therefore, the study of herbs at the compatibility level is of great significance in the clinical application of TCM, extending beyond the challenge of studying TCM prescriptions.

**FIGURE 1:**
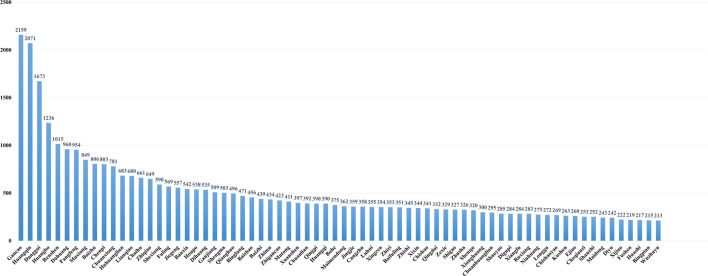
The frequency of *Coptidis rhizoma* combined with other herbs in the relative formulae (compatibility frequency ≥ 210).

*Coptidis rhizoma* (CR) was first mentioned in the Eastern Han Dynasty (25–220 AD) in *Shennong’s Classic of Materia Medica* (神农本草经). CR is bitter in flavor and cold in nature, and is attributed to the heart, spleen, stomach, liver, gallbladder, and large intestine meridians. It has the effect of clearing heat and drying dampness, purging fire, and detoxification. It is used for conditions such as hot and humid fullness, vomiting and acid swallowing, diarrhea, jaundice, high fever and dizziness, heart-fire hyperactivity, upset insomnia, palpitations, and so on. Additionally, it can also be used externally for eczema and wet sores. CR is one of the most commonly used herbs to purge Xin fire. Modern studies showed that CR and its main alkaloids have anti-atherosclerosis, anti-inflammatory, and antibacterial effects, among others (Meng et al., 2018). It is commonly used as a major herb for treating respiratory disorders, pediatric disorders, digestive disorders, dermatological disorders, and nervous system disorders. A presentation on the species of seven herbs in botany, phytochemistry, and pharmacology is summarized in Supplementary Table S1. In the clinical practice of TCM, many herbs are often combined with CR to form herb pairs. Through statistical analysis, it was found that there were 75 herbs (compatibility frequency ≥ 200), 30 herbs (compatibility frequency > 400), and even five herbs (compatibility frequency > 1000), namely, *Glycyrrhizae radix et rhizoma* (GRR), *Scutellariae radix* (SR), *Angelicae Sinensis radix* (ASR), *Phellodendri Chinensis cortex* (PCC), and GRER (Figure 1). We reviewed the key traditional uses, changes in bioactive constituents, and compatibility effects of several major herb pairs containing CR, particularly CR and *Euodiae fructus* (EF), SR, *Magnoliae Officinalis cortex* (MOC), GRR, *Ginseng radix et rhizoma* (GRER), and *Aucklandiae radix* (AR) have been recognized as herb pairs and have been relatively extensive studies. These six herb pairs are commonly used in clinical practice due to their pharmacological properties and clinical effects. This is the reason why we selected them for our study.

In recent years, research on herb pairs has gradually deepened, leading to remarkable achievements in theoretical summaries, efficacy substance analysis, compatibility effect evaluation, mechanism research, clinical application, and product development. CR is a widely used herb with significant medicinal value that deserves recognition. It has strong efficacy and is often utilized in the treatment of respiratory diseases, digestive diseases, pediatric diseases, skin diseases, and nervous system diseases (Wu et al., 2015). According to statistics, there are more than 32,000 types of prescriptions in 13 ancient prescription books before the Song Dynasty, about 5% of which mention CR. CR can be combined with a variety of herbs, and different herbal combinations can produce various effects, which is of great significance for the development of new drugs. The bitter and cold nature of CR can irritate the spleen and stomach, and certain ingredients in CR may trigger allergic reactions. Herb combinations can help alleviate the side effects caused by CR. It can be seen that the compatibility of CR is worthy of attention. CR is a heat-clearing medication commonly combined with Chinese traditional medicines like SR and PCC, which have similar effects, to enhance the heat-clearing and dampness-reducing effects. When combined with wind-cold-dispersing herbs such as *Saposhnikoviae radix* and *Perillae folium*, it can help relieve exterior syndrome and expel wind. The compatibility of CR with various herbs can alter its clinical indications, making it more appropriate for different diseases, and also modifying its pharmacodynamic effects. CR is included in various traditional Chinese formulas, such as *Gegen Qinlian* Decoction (GGQLD) and *Qingwei Huanglian* Pill. At the same time, certain medicinal pairs including CR have been incorporated into the Chinese Pharmacopoeia. For example, *Zuojin* Pill (ZJP) (Wei et al., 2021a), and XLP (Dai et al., 2023) are clinically used to treat gastrointestinal-related diseases. The objectives are aimed at clinical application. Currently, research on the material basis and related mechanisms of herb compatibility mainly focuses on pharmacodynamics, pharmacokinetics and Omics (Song et al., 2017). There is little research on the changes in chemical components before and after herb compatibility (Mu et al., 2024). Lack of in-depth research. Although some progress has been made in the research on CR and individual herb pairs, scholars often overlook the systematic investigation of whether there are changes in chemical components compared to single herbs in herb pairs, such as CR-GRER (Wu et al., 2021). Supramolecular technology and plant metabolomics technology are beneficial for analyzing the changes in chemical components before and after drug compatibility. Therefore, by combining these two aspects, we systematically reviewed the chemical composition, pharmacokinetics, pharmacodynamics, metabolomics, and other aspects of herb pairs before and after compatibility would be more conducive to new drug development and clinical application. In addition, modern research on traditional herb pairs is not comprehensive, especially lacking in association rules, data mining research on pairs, and changes in the chemical composition of herbs before and after compatibility. Exploring the compatibility rules of herb pairs and fully tapping into their intrinsic value for clinical theory is crucial. CR is a representative drug for clearing heat and removing toxins. An overview of commonly used herb pairs containing CR is conducted through a literature review and relevant data analysis, followed by a discussion of current research progress and prospects. This provides a reference for modern clinical prescriptions in TCM and guides the modern research and development application of these herb pairs.

## 2 Materials and methods

A thorough search for studies on herb pairs containing CR was conducted from PubMed, Elsevier, Web of Science, Library Genesis, and CNKI. Additionally, information was obtained from local books, doctoral and master’s theses, and pharmacopeias. The data about the herb pair containing CR has been compiled. The keywords used include *Coptidis rhizoma*, CR, Huanglian, traditional uses, herb pairs, their combinations, and others. Chemical structures were drawn using ChemDraw 20.0 software.

## 3 Main herb pairs containing *Coptidis rhizoma*

**FIGURE 2:**
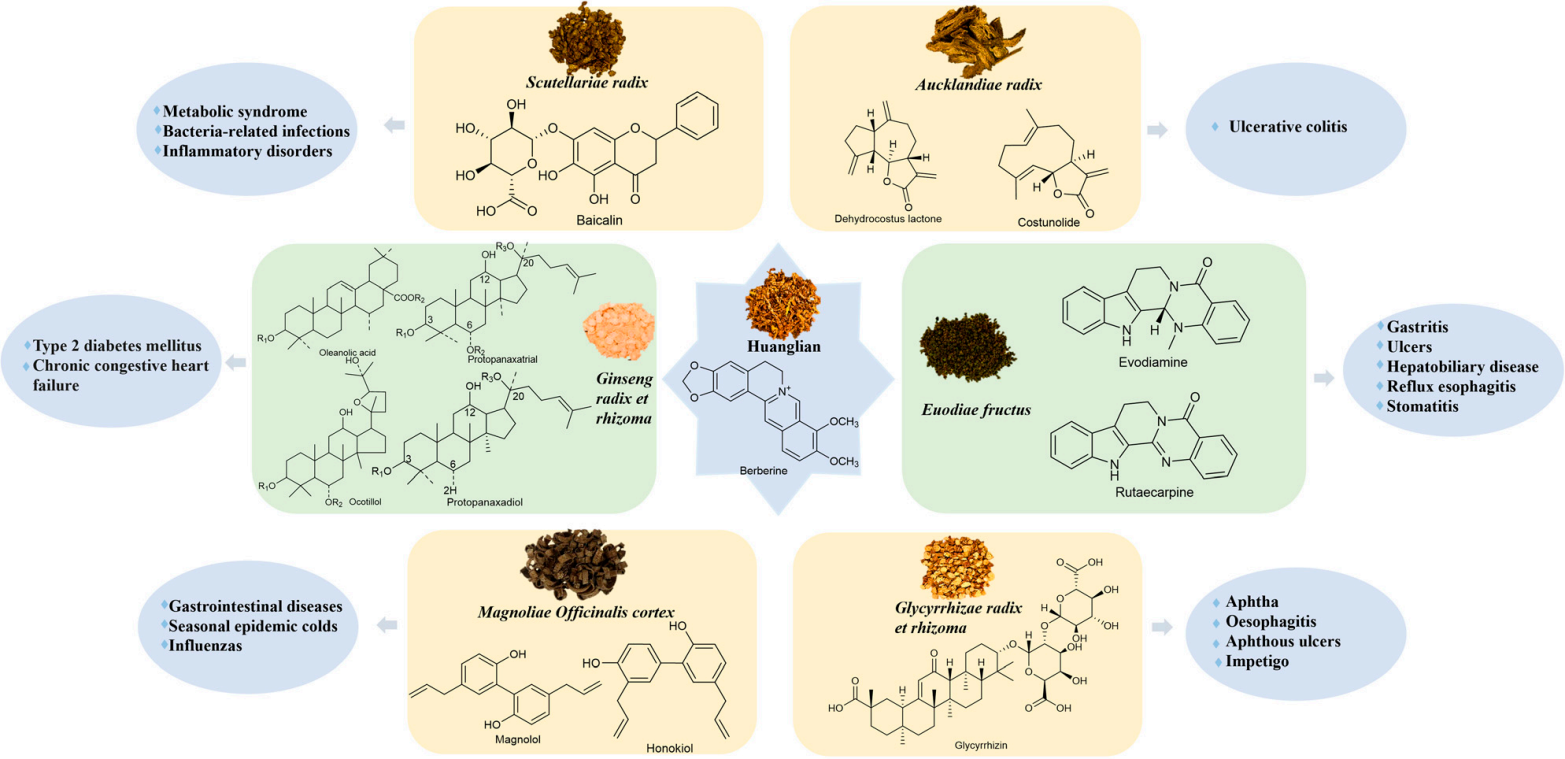
The correlation between the main chemical components and clinical applications of *Coptidis rhizoma* and other herbs, respectively.

CR is a well-known herb in the world. The three mentioned above are known as “Wei Lian,” “Ya Lian,” and “Yun Lian” respectively. CR is referred to as “Guo Lao,” which means “a national treasure plant.” There are more than one hundred chemical components that can be isolated from CR, including alkaloids, lignans, and acidic components, among which alkaloids are the most abundant and the primary sources of bioactive constituents. Therefore, we concluded the structures of alkaloids that can be found in references in Supplementary Table S2. Among them, berberine is the primary bioactive ingredient (Yi et al., 2013). It is commonly used in combination with other herbs such as EF, SR, MOC, GRR, GRER, and AR. The correlation between the main chemical components and clinical applications of CR and the other six herbs, respectively, is shown in Figure 2. We have tallied the herb pairs that have been researched by scholars and have been combined in prescriptions more than 200 times (Table 1).

**TABLE 1:** The compositions, frequency, traditional uses, and related books of herb pairs containing *Coptidis rhizoma* (compatibility frequency > 200).

NO.	Herb pairs	Latin name	Frequency	Traditional and clinical uses	The related recorded books
1	Huanglian and Gancao	*Coptidis* *rhizoma*, *Glycyrrhizae* *radix* *et* *rhizoma*	2159	Clearing heat and drying dampness, purging fire and detoxification. diabetes	*Doumen Fang* (斗门方)
2	Huanglian and Huangqin	*Coptidis* *rhizoma*, *Scutellariae* *radix*	2071	Clearing heat, drying dampness, and purging fire. Diabetes	*Treatise on Cold Damage Disorders* (伤寒论)
3	Huanglian and Huangbo	*Coptidis* *rhizoma*, *Phellodendri Chinensis* *cortex*	1236	Clearing heat, drying dampness, detoxifying	*Treatise on Cold Damage Disorders*
4	Huanglian and Renshen	*Coptidis* *rhizoma*, *Ginseng* *radix* *et* *rhizoma*	1015	Nourishing Qi and Yin, clearing heat and drying dampness	*Curative Measures for All Diseases* (万病回春)
5	Huanglian and Dahuang	*Coptidis* *rhizoma*, *Rhei* *radix* *et* *rhizoma*	960	Purging fire, drying dampness	*Treatise on Cold Damage Disorders*
6	Huanglian and Muxiang	*Coptidis* *rhizoma*, *Aucklandiae* *radix*	849	Clearing heat and drying dampness, relieving pain, and moving pain	*Prescriptions of the Pharmacy Bureau*
7	Huanglian and Lianqiao	*Coptidis* *rhizoma*, *Forsythiae* *fructus*	680	Purging fire and detoxification, dispersing swelling and detumescence	*Orthodox Manual of External Diseases* (外科正宗)
8	Huanglian and Banxia	*Coptidis* *rhizoma*, *Pinelliae* *rhizoma*	542	Clearing heat and phlegm, clearing knot and stopping vomiting	*Treatise on Cold Damage Disorders*
9	Huanglian and Houpo	*Coptidis* *rhizoma*, *Magnoliae Officinalis* *cortex*	538	Dispelling cold and clearing away cold	*Taiping Holy Prescriptions for Universal Relief*
10	Huanglian and Dihuang	*Coptidis* *rhizoma*, *Rehmanniae* *radix*	535	Diabetes	*Supplement to the Essential Prescriptions Worth a Thousand Gold*
11	Huanglian and Shengma	*Coptidis* *rhizoma*, *Cimicifugae* *rhizoma*	503	Clearing stomach fire and detoxifying	*Supplement to the Essential Prescriptions Worth a Thousand Gold*
12	Huanglian and Binglang	*Coptidis* *rhizoma*, *Arecae* *semen*	471	Clearing heat and drying dampness	*General Records of Holy Universal Relief* (圣济总录)
13	Huanglian and Mutong	*Coptidis* *rhizoma*, *Akebiae* *caulis*	411	Clearing heart fire	*Confucians’ Duties to Their Parents* (儒门事亲)
14	Huanglian and Zhizi	*Coptidis* *rhizoma* *, Gardeiae* *fructus*	353	Enteritis	*Arcane Essentials from the Imperial Library* (外台秘要)
15	Huanglian and Zhishi	*Coptidis* *rhizoma*, *Aurantii* *fructus* *immature*	345	Clearing heat and purging fire, damp-heat diarrhea, Dysentery	*Neiwai Shangbian Huolun* (内外伤辨惑论)
16	Huanglian and Kushen	*Coptidis* *rhizoma*, *Sophorae Flavescentis* *radix*	263	Clearing heat and drying dampness, Colitis	*Prescriptions for Universal Relief*
17	Huanglian and Ejiao	*Coptidis* *rhizoma*, *Asini Corii* *colla*	260	Insomnia	*Treatise on Cold Damage Disorders*
18	Huanglian and Wuzhuyu	*Coptidis* *rhizoma* *, Euodiae* *fructus*	213	Clearing liver fire, Gastritis	*Taiping Holy Prescriptions for Universal Relief*

### 3.1 *Coptidis rhizoma* and *Euodiae fructus*

#### 3.1.1 Traditional uses of *Coptidis rhizoma* and *Euodiae fructus*

EF is hot in nature, pungent and bitter in flavor, and slightly toxic. It has the effects of dispersing cold and relieving pain, lowering rebelliousness and stopping vomiting, and is often used to treat headaches, dermatophytosis, gastric ulcers, menorrhagia, aphtha, and emesis (Chinese Pharmacopoeia Commission, 2020). One of the most classic and clinically used medicinal pairs of CR-EF is the *Zhuyu* Pill (ZYP) in the *Taiping Holy Prescriptions for Universal Relief* (太平圣惠方) of the Song Dynasty. However, ZJP is the most representative formula from *Danxi’s Mastery of Medicine* (丹溪心法, Yuan Dynasty). Zhao et al. (2009) studied the differences in the effects of ZJP and its similar formulations on the Wei cold model in rats. The results indicated that ZJP, which consists of CR and EF in a ratio of 6:1, has better therapeutic effects on Wei heat syndrome. This is due to the larger proportion of CR, which has a cold property. Fan-ZJP, made of CR and EF in a ratio of 1:6, is suitable for treating Wei cold syndrome due to the high proportion of EF, which has a hot property. *Ganlu* Power (GLP) and ZYP, composed of CR and EF in proportions of 2:1 and 1:1, respectively, have been used to treat summer qi diseases and deficient cold dysentery because the ratio of hot and cold herbs located in the middle of ZJP and Fan-ZJP.

#### 3.1.2 Bioactive components of the *Coptidis rhizoma*-*Euodiae fructus* herb pair

##### 3.1.2.1 Bioactive component variation of the *Coptidis rhizoma*-*Euodiae fructus* herb pair

Evodiamine and rutaecarpine are the main active components of EF (Li et al., 2021). A study on the major chemical components of the CR-EF herb pair in different ratios (1:0, 6:1, 3:1, 2:1, 1:1, 1:2, 1:3, 1:6, 0:1) showed that one of the eight components was not detected. The fraction may be a new substance produced by the combined decoction. In addition, some studies have shown that the content of alkaloids in CR decreased to different degrees after being combined with EF. With the increase in the proportion of EF, the content of alkaloids in CR decreased significantly (Ye et al., 2000). This may be due to the fat-soluble components of CR having a solubilizing effect on the fat-soluble alkaloids in EF during the decoction process (Jiang et al., 2014).

##### 3.1.2.2 Bioactive component variation of the *Coptidis rhizoma*-*Euodiae fructus* herb pair *in vivo*

Alkaloids, as the main active substances in CR and EF, have been extensively studied for their pharmacokinetics. ZJP is a traditional Chinese medicinal formula that combines two herbs, CR and EF, in a ratio of 6:1 (Zhao et al., 2010). A study by Yan et al. (2011) found that the t_max_ after administering ZJP is longer compared to CR, the C_max_ after administering ZJP is lower than that of CR, and the AUC decreases after combining CR and EF. Deng et al. (2008) found that the pharmacokinetic profiles of berberine, jateorrhizine, and palmatine after the administration of ZJP showed similar three peaks, possibly due to their molecular structures. Deng reviewed the distribution of protoberberine after oral administration of ZJP, and the results suggested that re-absorption and enterohepatic circulation may be responsible for the multiple blood concentration peaks. Meanwhile, a growing body of research has revealed that the ADME feature of this herb pair exhibits inconsistency at different compatibility ratios. For instance, CR-EF (6:6) was most effectively absorbed in the rat jejunum, but CR-EF (2:1) was effectively absorbed in the ileum. Moreover, according to the current study, berberine had better absorption, a longer half-life, and a shorter peak time in ZJP when compared to other combinations like 2:1 and 1:1 (Tu et al., 2011). To summarize, the interactions between CR and EF cause significant changes in the pharmacokinetic profiles of dehydroevodiamine and coptisine. CR can enhance the effect of EF, while EF can weaken the effect of CR. The connotation of prescription is so complex that it is difficult to elucidate solely through qualitative analysis.

#### 3.1.3 Compatibility effects of the *Coptidis rhizoma*-*Euodiae fructus* herb pair

EF is often combined with other herbs to treat digestive, cardiovascular, gastrointestinal, and gynecologic diseases. The CR-EF herb pair has pharmacological effects of antibacterial, antioxidant, and anti-inflammatory properties (Li and Wang, 2020). It has been extensively utilized in clinical practice. Various ailments require different proportions and combinations, commonly used in 2:1, 1:1, 6:1, 1:6, or other combinations, primarily for treating liver and gastrointestinal diseases (Tong et al., 2021a; Yu et al., 2021). To treat nonalcoholic fatty liver disease, the CR-EF herb pair is typically used in a ratio of 2:1 (Zhang et al., 2022). In addition, when the ratio of CR and EF is 8:3, the combination of CR and EF can be used to treat abdominal mass (Su et al., 2023). We summarized the compatibility effects, ratio, and efficacy of the CR and EF herb pair as follows.

##### 3.1.3.1 Effects on the gastrointestinal diseases

**FIGURE 3:**
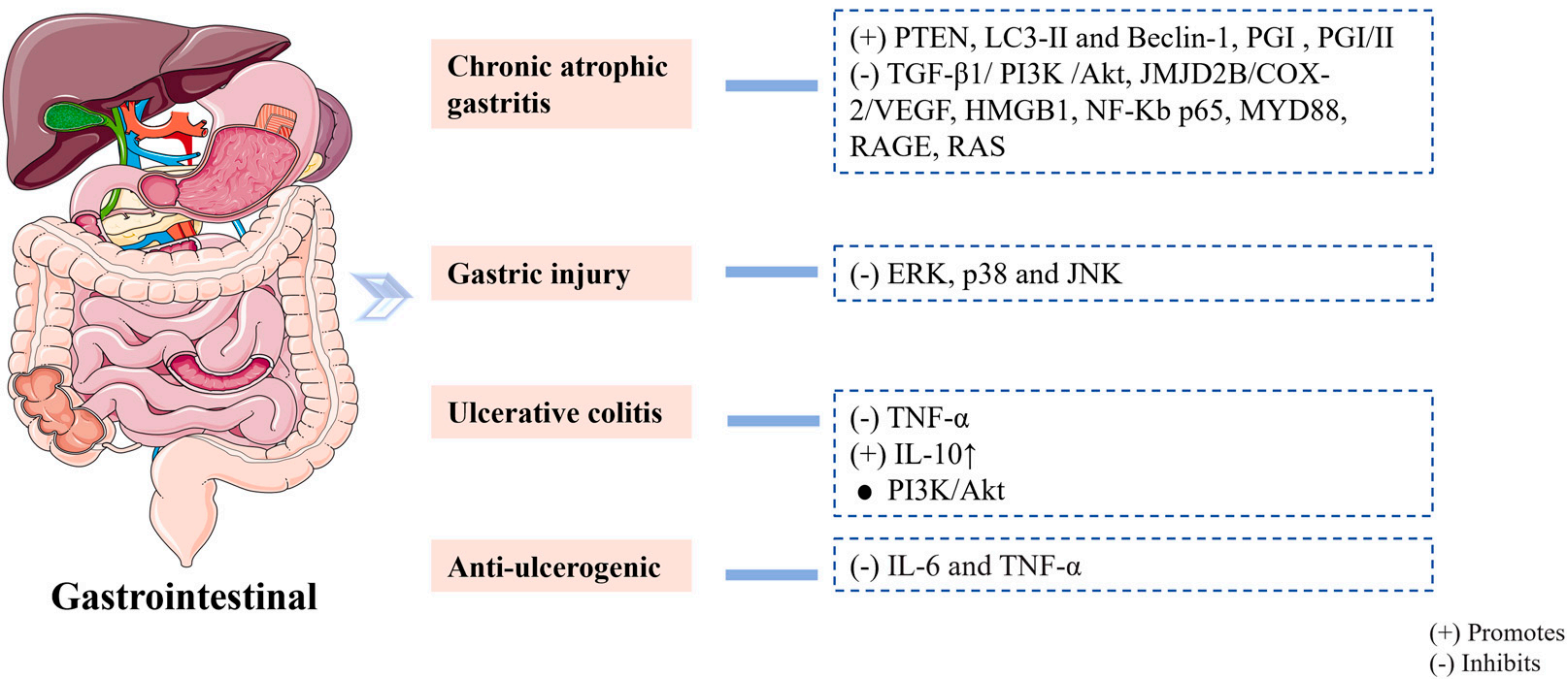
The mechanisms of the *Coptidis rhizoma*-*Euodiae fructus* herb pair against gastrointestinal diseases.

CR-EF, with a ratio of 6:1, has been used in China for a long time to treat gastrointestinal diseases, especially chronic atrophic gastritis, gastric injury, acute gastric lesions and ulcerative colitis (UC) (Figure 3). Many studies have shown that ZJP can treat chronic atrophic gastritis mainly by improving gastric histomorphology and gastric tissue inflammatory lesions (Tong et al., 2021a; Tong et al., 2021b; Wen et al., 2022). Additionally, it can alleviate gastric injury by ameliorating the inflammatory infiltration of gastric tissue (Wei et al., 2021a). In Asia, ZJP has been used clinically for centuries to treat gastritis and gastric ulcers. The mechanism of action for the two herbs in preventing ulcers may mainly involve the regulation of inflammatory cytokines to protect the gastric mucosa (J. Wang et al., 2015). The most significant effect on UC was seen with the CR-EF herb pair. Wei et al. (2021b) explored the bioactive ingredients of ZJP in the treatment of UC. The results demonstrated that the predicted active ingredients of ZJP may be effective in treating UC by regulating the MAPK and PI3K-Akt pathway. Zhou et al. (2020) studied the effect of ZJP on correlation with gut microbiota and Treg cells in DSS-induced colitis, and the results indicated that ZJP controls the interaction between intestinal microflora and Treg cells to reduce the effects of UC. Meanwhile, Cai et al. (2022) indicated that ZJP can reduce DSS-induced rat UC by crosstalk between host metabolites and gut microbiota. Furthermore, Luo et al. (2019) investigated the differences in the immunomodulatory activities of ZJP and Fan-ZJP. The main difference was that ZJP primarily reduced the level of IL-1β, while Fan-ZJP increased the level of IL-10 in serum. These results help us understand the distinction between the effects of ZJP and Fan-ZJP on functional gastrointestinal disease models. It is related to the properties of cold and heat and provides a foundation for the rational clinical utilization of the CR-EF herb pair.

##### 3.1.3.2 Anti-tumor effect

**FIGURE 4:**
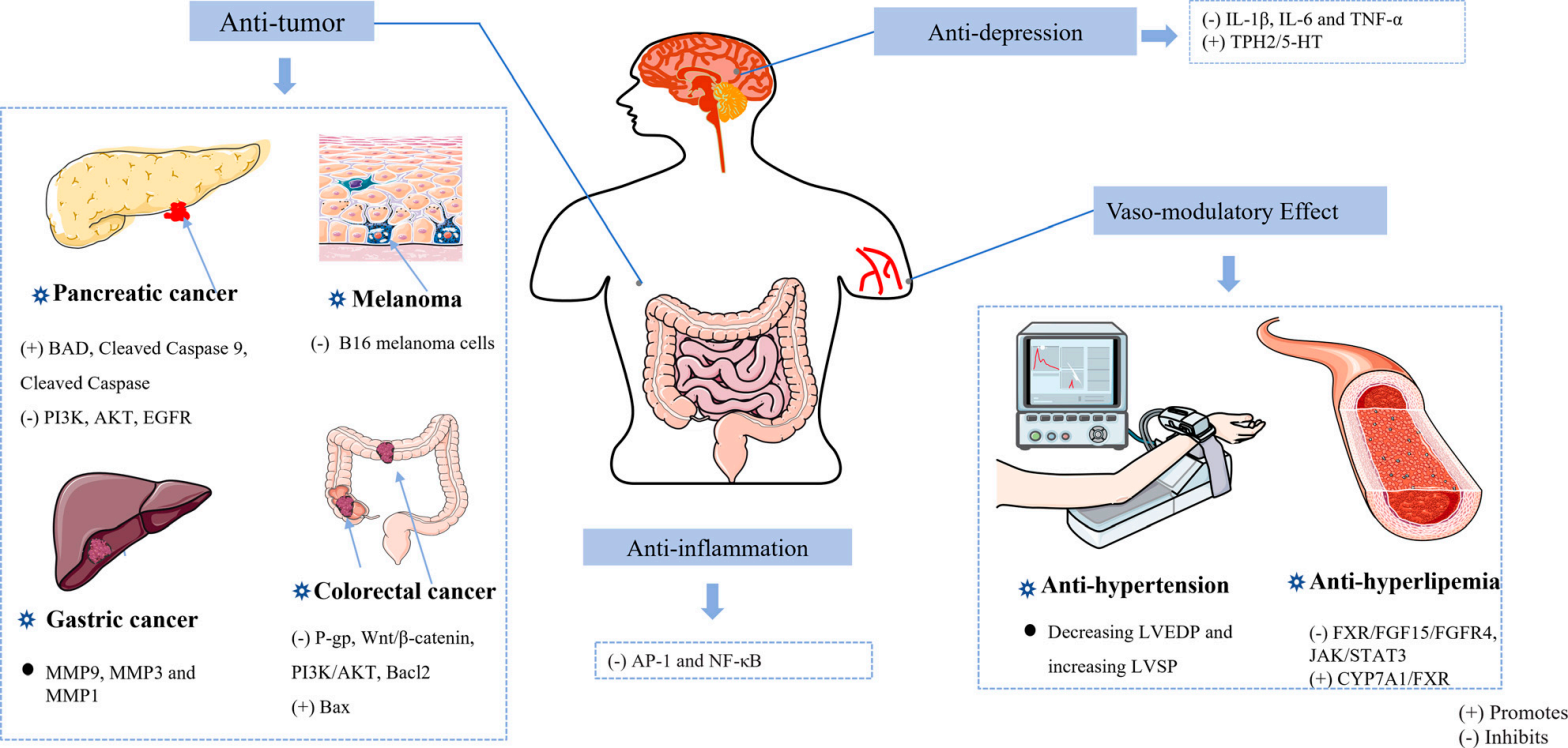
The mechanisms of the *Coptidis rhizoma*-*Euodiae fructus* herb pair against tumors, depression, hypertension, hyperlipemia, and inflammation.

At the moment, the most significant disease endangering human health is cancer. ZJP could potentially serve as an anti-tumor agent. A growing number of findings indicate that herbs exert their pharmacological effects through multiple targets and pathways (Figure 4). ZJP can inhibit pancreatic cancer by acting on JUN, TP53, and MAPK1 targets. Moreover, the results of Vitro experiments indicated that ZJP can prevent pancreatic cancer by inhibiting the apoptosis of pancreatic cancer cells (Wang et al., 2021). Fan et al. (2022) indicated that the main components of ZJP can effectively target specific genes to fight against colorectal cancer. The combined effects of evodiamine and berberine demonstrated synergistic anti-colorectal cancer (Guan et al., 2020). Additionally, some research indicated that ZJP can treat colorectal cancer by inducing apoptosis of HCT116 cells and inhibiting cell migration and invasion (S. Huang et al., 2020; Pan et al., 2017). Overall, we can treat tumors by looking for drugs to inhibit the metastasis and proliferation of tumor cells, such as S180 cells, HepG2 cells, and SGE-7901 cells (Wang et al., 2009; Chao et al., 2011). Zhang et al. (2020) determined a possible pharmacological mechanism of ZJP in treating gastric cancer (GC), and the results suggested that some matrix metalloproteinase family members may play a role in ZJP’s anti-gastric cancer action. Besides, some studies have reported that both berberine chloride and evodiamine have anti-melanoma effects (Lin et al., 2020). Overall, the combination of CR and EF in a ratio of 6:1 has been found to have significant inhibitory effects on various cancers.

##### 3.1.3.3 Anti-hypertensive and anti-hyperlipidemia effects

Studies have shown that the CR-EF herb pair significantly affects hypertension and hyperlipemia (Figure 4). A study has shown that ZJP has definite anti-hypertensive effects (Bai, 2009). Chu et al. (2008) showed that the combination of CR and EF in a ratio of 3:1 can significantly decrease left ventricular ejection fractions and elevate left ventricular systolic pressure in hypertensive rats, thus enhancing cardiac systolic and diastolic functions. The mechanism of the anti-hypertensive effect may be associated with the repair of oxidative cell damage Wang, Chen et al. (2019). Shen (2007) studied the anti-hyperlipidemia effects of various ratios (6:1, 2:1, 1:1) of CR and EF, the most effective anti-hyperlipidemia effect was observed when the ratio of CR and EF was 1:1. Zhou et al. (2018) studied the effective components berberine and evodiamine in CR and EF, and the results showed that the combination of CR and EF components may play a role in lowering lipids by improving liver adiposity and decreasing the gene expression of the JAK2/STAT3. Another study showed that the combination of CR and EF in a ratio of 1:1 plays a role in lipid-regulating effects by regulating the FXR/FGF15/FGFR4 pathway (Xu, 2020).

##### 3.1.3.4 Antipyretic effect

The CR-EF herb pair is also useful for antipyretics and heatstroke. Gao et al. (2010) investigated the herb pair CR-EF and found that different ratios of CR and EF (6:1, 2:1, 1:1, 1:6) could reduce rats’ rectal temperature and mortality rate to varying degrees and prolong their survival time. Among them, the anti-heat stroke effect is the most significant when the ratio of CR and EF is 2:1. A similar study by Zhang et al. (2017).

##### 3.1.3.5 Anti-inflammation and anti-depression effect

Depression is a prevalent and debilitating illness that is chronic and recurrent, with common symptoms being slowed thinking and reduced volitional activity (Singla et al., 2021). According to several studies, ZJP can effectively protect against Ang II-induced endothelial inflammation by regulating NF-κB signaling pathway (Ko et al., 2007). GLP can treat nonalcoholic steatohepatitis by regulating AKT1 (Gao et al., 2022). Furthermore, it was also indicated that the ethanol extract of ZJP could inhibit the expression of inflammatory mediators in lipopolysaccharide-stimulated 264.7 mouse macrophages. The anti-inflammatory effect of ZJP may be related to NF-κB, while the antidepressant activity may be associated with 5-HT neurons (Wang et al., 2012). Furthermore, studies have demonstrated that ZJP can improve depression-like behavior via the TPH2/5-HT pathway or regulate the levels of neurotransmitters and inflammatory cells (Wang et al., 2013; Wang et al., 2020; Wang et al., 2023). Although many antidepressants can alleviate depressive symptoms, the side effects cause many depressed patients to respond unsatisfactorily to commercially available antidepressants. Therefore, developing effective, safe, and low-side-impact antidepressant drugs has become the focus of current research. Today, traditional herbs are a better option for treating depression.

#### 3.1.4 Conclusion on *Coptidis rhizoma*-*Euodiae fructus*

The CR-EF herb pair plays an important role in current clinical applications as a TCM formula. Different ratios of the two can show different or even contradictory effects. At present, the chemical composition and pharmacological effects of CR and EF have been well studied. However, there is a lack of research on the correlation between the two. With the rapid development of modern Chinese medicine pharmacodynamic substance identification technology, it is important to further enhance the correlation between the components of the pairs and their efficacy. This will help clarify the pharmacodynamic substances of the pairs. Based on existing analyses, the pharmacological mechanism of CR-EF was studied in depth using modern pharmacological and molecular biological techniques to reveal its mechanism of action at the molecular level. This research will provide a scientific basis for further clinical applications.

### 3.2 *Coptidis rhizoma* and *Scutellariae radix*

#### 3.2.1 Traditional uses of *Coptidis rhizoma-Scutellariae radix*

SR is bitter in flavor and flat in nature, and has the functions of clearing heat, moistening dryness, purging fire, and detoxification. SR is used in treating chest tightness and vomiting, damp-heat lumps and fullness, diarrhea, jaundice, lung-heat cough, high fever, and thirst (Chinese Pharmacopoeia Commission, 2020). The CR-SR herb pair is derived from the Treatise on Cold Damage Disorders and is used to alleviate heat, dryness, and dampness. It is commonly used to treat symptoms such as eye redness and swelling, mouth sores, and tongue inflammation caused by high fever and irritability. There are records of the CR-SR herb pair in *Banxia Xiexin* Decoction, *Huanglian Jiedu* Decoction (HLJDD), *Sanhuang* Decoction, and others.

#### 3.2.2 Bioactive components of *Coptidis rhizoma-Scutellariae radix*

##### 3.2.2.1 Bioactive component variation of *Coptidis rhizoma*-*Scutellariae radix* herb pair

Baicalin is the main active component of SR (Wei et al., 2018), which is widely used in TCM due to its diverse pharmacological effects, including antioxidant, anti-diabetic, anti-viral, and anti-ulcer properties (Baradaran Rahimi et al., 2021). The compatibility efficacy of CR and SR are related to changes in their essential substances. Some results suggested that the chromatographic peaks of the compatibility between CR and SR exhibited additive characteristics and generated new chromatographic peaks. The content of berberine and baicalin in the aqueous extract of the herb pair is significantly higher than that of a single herb. Therefore, berberine and baicalin can be extracted more effectively by co-decoction, which further confirms the synergistic effect of combining CR and SR. The content of baicalin is highest when the ratio of CR and SR is 2:1 (Wu et al., 2016). Baicalin and berberine are the main bioactive components in CR and SR, respectively, which were identified in the precipitate and formed a berberine-baicalin complex. The precipitate contains chemical components that have physiological effects. In addition, the study showed that the antibacterial activity of the precipitate complex was stronger than that of SR extract but weaker than that of berberine (Zhang et al., 2014).

##### 3.2.2.2 Bioactive component variation of *Coptidis rhizoma*-*Scutellariae radix* herb pair *in vivo*

The pharmacokinetics of a single component do not accurately represent traditional herbs due to the complex and numerous constituents they contain. Pharmacokinetics and pharmacodynamics are key factors in evaluating the efficacy of traditional herbs and explaining their mechanism (Ding et al., 2016). Some studies have shown that the concentration-time curves of baicalin and berberine in rat plasma exhibit double peaks. This indicated that these two substances may have enterohepatic circulation in rats. Enterohepatic circulation in pharmacodynamics has shown that the effect of the herb is significantly prolonged (He et al., 2014). The key processes influencing pharmacokinetics are absorption and metabolism, and the interaction between CR and SR needs further investigation.

#### 3.2.3 Compatibility effects of *Coptidis rhizoma-Scutellariae radix*

**FIGURE 5:**
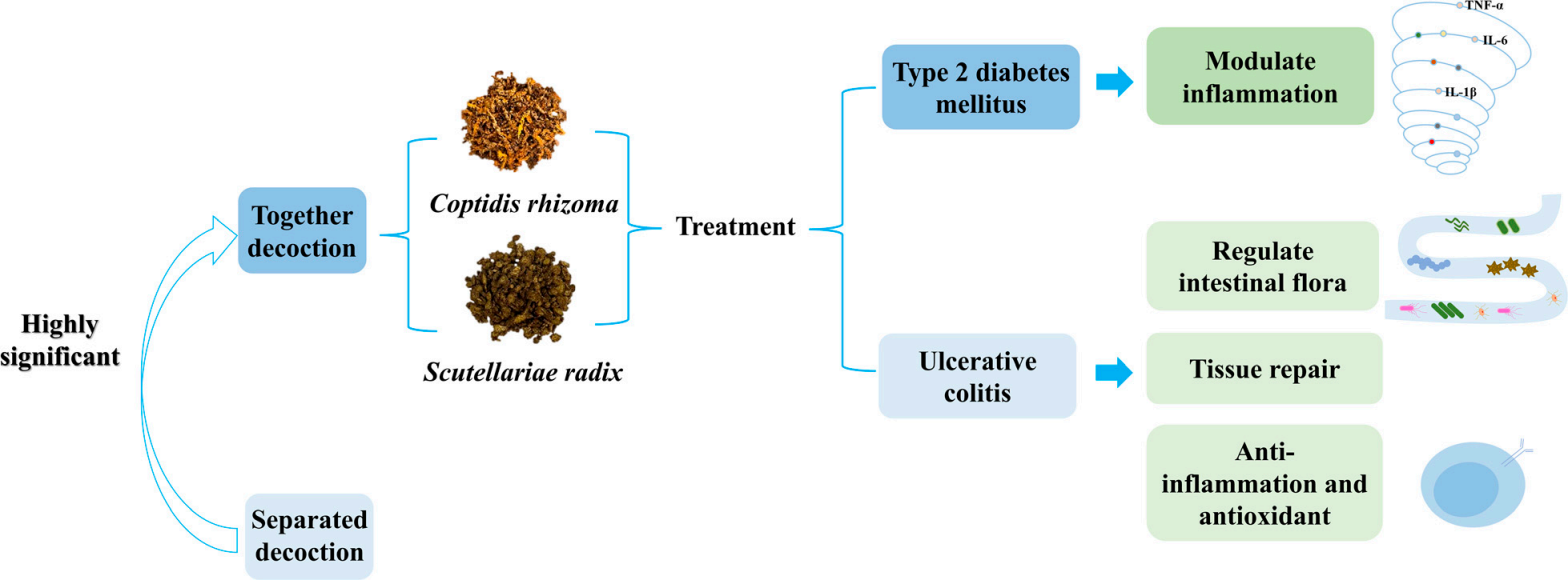
*Coptidis rhizoma*-*Scutellariae radix* can treat T2DM and UC, and the effect is more significant than that of a single herb.

Previous studies have summarized over 200 common TCM therapies for treating diabetes from nine classical works, such as *Essential Prescriptions Worth a Thousand Pieces of Gold* (备急千金药方). Yan showed that the combination of baicalin and berberine hydrochloride ameliorates colitis through maintaining the balance of proinflammation cytokines and anti-inflammation cytokines (Yan et al., 2022). In China, CR and SR are often combined in a 1:1 ratio for the treatment of diabetes mellitus. Baicalin and berberine exhibited an additive effect at low dosages, but they demonstrated antagonistic behavior at higher doses of baicalin. A study showed that the combination of CR and SR can improve T2DM rats by regulating the MAPK/PI3K/Akt pathway (Cui et al., 2018). CR or SR significantly improved the gut microbiota composition and glycolipid metabolism of T2DM rats (Xiao et al., 2020). In particular, when CR was combined with SR, the effect was more powerful (Liu et al., 2022) (Figure 5). Some studies have demonstrated that the ratio of SR and CR had a substantial synergistic impact when it was 3:2 or 1:3. When the ratios were adjusted to 1:1, they exhibited the most synergistic effect compared to any other percentage. In terms of specific doses, experts suggest that the treatment of T2DM with SR and CR should follow the principle that serious diseases require strong medicine. The recommended dosage of SR ranges from 9 to 15 g, while the dosage of CR should be 9–30 g. In some cases, the dosage of CR can be increased to 15–30 g (X. Huang et al., 2020). Additionally, the extract of the SR-CR herb pair and its primary constituents play a therapeutic role in UC and hyperglycemia (Liu et al., 2013). Additionally, some studies have revealed that CR can inhibit the absorption and metabolism of flavonoids, indicating a significant interaction between herbs. Most of the compatibility between CR and SR indicated synergistic effects, with some exhibiting additive effects and a few showing apparent antagonistic effects.

#### 3.2.4 Conclusion on *Coptidis rhizoma-Scutellariae radix*

The CR-SR pair has an important place in TCM. At present, the CR-SR herb pair is often used in a ratio of 1:1 to treat diabetes mellitus. There is a lack of research on changes in dissolving bioactive ingredients and their clinical applications at different compatibility ratios. Therefore, further studies are needed to establish a solid foundation for exploring additional applications. In addition, further investigation is needed to determine the optimal ratio of CR-SR. From the perspective of modern pharmacology, the compounds found in CR and SR exhibit a range of biological activities, including antioxidant, anti-inflammatory, antibacterial, antiviral, and antitumor effects. Modern scientific and technological methods can be used to explore their roles in the treatment of diseases. Therefore, integrating traditional applications with modern pharmacological effects can enhance the therapeutic efficacy of the CR-SR pair and facilitate their extensive utilization in clinical applications.

### 3.3 *Coptidis rhizoma* and *Magnoliae officinalis cortex*

MOC is bitter and acidic in flavor and warm in nature. It can be applied in the treatment of abdominal distention, vomiting, diarrhea, food accumulation, Qi stagnation, constipation, phlegm and fluid retention, and cough resulting from asthma (Chinese Pharmacopoeia Commission, 2020). Honokiol and magnolol are the predominant active constituents of MOC, which have many pharmacological effects and play an indispensable role in China’s medical care (Niu et al., 2021). Except for magnolol, all of the components have been documented to have the potential to treat UC (Chen et al., 2019).

MOC is often combined with other Chinese herbs and is not usually used alone, such as *Houpo Sanwu* Decoction, *Huoxiang Zhengqi* Powder, *Houpo Mahuang* Decoction, and *Banxia Houpo* Decoction (Zhang, 1997). *Huanglian Houpo* Decoction (HHD) is recorded in the Chinese medicine monograph *Prescriptions for Universal Relief* (普济方) in the Ming Dynasty (1390 A.D.), which combines CR and MOC in a 1:1 ratio. It was frequently used to treat ulcers, seasonal epidemic colds, diarrhea, and influenza. When CR and MOC were in different proportions, the content of honokiol and magnolol differed. This may be due to berberine being a quaternary alkaloid, while honokiol is a biphenolic constituent. Furthermore, when they act together, honokiol promotes the absorption of berberine. According to a specific study, the concentration of berberine in HHD extracts in rat plasma is significantly higher than that of the CR extract (Meng and Zhang, 2009). Berberine hydrochloride and magnolol are considered to be the two main anti-influenza components of CR and MOC, respectively. Some studies have shown that HHD inhibits H1N1 infection *in vivo* and effectively treats influenza-induced pneumonia in mice by reducing the levels of TLR3, TLR7, MYD88, and NF-κB p65 (Zhang et al., 2021). Data analysis indicates that HHD has a significant therapeutic effect on UC (Cheng et al., 2023). Compared to CR and MOC alone, combining the two herbs in a ratio of 3:2 can prevent damage to the intestinal barrier, resulting in a more significant outcome (Wang et al., 2022). Moreover, when CR is combined with MOC, it can enhance the antidiarrheal effect of CR. At the same time, it can alleviate the bitter and cold nature of CR (Xie et al., 2022). It can be seen that the protective effect of CR and MOC on the gastrointestinal tract involves many factors. Most of the experimental studies on CR and MOC for the treatment of UC have focused on their single herb or main components. However, there is little research on the combination of CR and MOC in treating UC, and the specific rules for their combination have not been identified. Therefore, it is essential to consider the optimal dosage to achieve synergistic benefits in treating UC. It is also possible to explore its application in disease prevention and other areas to further expand its clinical applications.

### 3.4 *Coptidis rhizoma* and *Glycyrrhizae radix et rhizoma*

GRR is flat in nature and sweet in flavor, and has the effects of tonifying the spleen and qi, moistening the lungs and relieving cough, clearing away heat and detoxifying, and reconciling various medicines (Chinese Pharmacopoeia Commission, 2020). It is known as the “Ancestor of Plants” and is widely used around the world. It was used to treat asthma, hoarseness, coughs, and lung diseases (Fiore et al., 2005). CR is combined with GRR in various well-known TCM formulations, such as the *Huanglian* Decoction, GGQLD (Ma et al., 2023), *Shengjiang Xiexin* Decoction (Guan et al., 2017), and *Banxia Xiexin* Decoction (Yi et al., 2023), which are detailed in the classic TCM book, *Treatise on Cold Damage Disorders*.

Nowadays, it is commonly used as a spice due to its flavor and medicinal value. GRR is primarily used as an adjuvant and mediator in herb pairs (Liu et al., 2015). Glycyrrhizin is the most significant component of GRR (Wang et al., 2015). When CR and GRR are combined, the glycyrrhizin reacts to form berberine glycyrrhizate salt, which will make the bitter taste of berberine disappear and play a role in correcting the taste. The precipitation slowly decomposes in the body and exerts its effects, which plays a role in moderating and prolonging the efficacy of herbs (Zhang et al., 2020). On the one hand, when comparing the retention times of chemical components in the chromatograms of single herbs and herb pairs through qualitative analysis, the results indicated that the changes in chemical constituents in the compound were not simply additive. This could be explained by the formation of new substances. On the other hand, when using the peak area normalization method to conduct a quantitative analysis of all components, the combination of CR and GRR could gradually release berberine. This compound has choleretic, anti-anemia, anti-cancer, and anti-radiation effects. It ensures the therapeutic effect of berberine and avoids the adverse impact of inhibiting Yang and injuring the spleen and stomach, which further verifies the rationality of the compatibility of CR and GRR (Zhao, 2008).

One of the most significant features of Chinese herbal medicine is multi-herb therapy. However, due to the complex composition of Chinese traditional decoctions, the substance that produces its therapeutic effect has not been fully identified. CR and GRR could form spherical supramolecules after decoction. These supramolecules enhance the ability of berberine to inhibit *Staphylococcus aureus* (Z. Wang et al., 2023). We believe that the supramolecular compounds in the co-decocted decoction of the CR-GRR herb pair are the main material basis for the antibacterial effect. A study demonstrated that the co-administration of glycyrrhizin and berberine exhibited more efficiency than berberine or glycyrrhizin administered alone. The combination of glycyrrhizin and berberine can attenuate ischemia-reperfusion injury by inhibiting the HMGB1/TLR4 pathway (Zhu et al., 2018). CR combined with GRR in a ratio of 3:2 has an anti-diarrheal effect. GRR can enhance CR’s effect (Yu et al., 2005). By analyzing the data of CR pairs, the results indicated that the most commonly used pairing was CR-GRR, with a frequency of 2159. However, there is a lack of modern studies on this topic. The optimal ratio of herb pairs, the variation of bioactive components *in vivo* and *in vitro*, and the effects of the combination need to be further studied. Future studies can explore its pharmacological mechanism, pharmacodynamic evaluation, and multidisciplinary joint application in greater depth to provide a scientific basis for its wider clinical application.

### 3.5 *Coptidis rhizoma* and *Ginseng radix et rhizoma*

GRER is sweet, slightly bitter in flavor, and slightly warm in nature, and has the functions of reinforcing the spleen and lungs, nourishing body fluids and blood, tranquilizing the mind, and benefiting intelligence (Chinese Pharmacopoeia Commission, 2020). Ginsenosides are the primary active ingredients in GRER and the main constituents used to treat diabetes and other chronic complications (Kim et al., 2017). *Huanglian Renshen* Decoction (HRD) is a classic traditional Chinese recipe composed of two herbs, CR and GRER, in a 1:1 ratio. The combination of CR and GRER to treat diabetes (known as Xiaoke in ancient times) can be traced back to *Essential Prescriptions Worth a Thousand Pieces of Gold* of the Tang Dynasty. The antioxidant activity of CR-GRER in the ratio of 5:8 was higher than that of the single decoction of CR and GRER. This difference may be attributed to the interaction between different substances of the two herbs during decoction (Du et al., 2010). CR is the most famous heat-clearing herb, and GRER is the most renowned Qi-supplementing herb. The combination of these two herbs makes HRD a representative and fundamental formula for treating Qi-Yin deficiency and excessive dry-heat syndrome, which is suitable for the treatment of diabetes. Therefore, it is widely used to treat T2DM in China and other Asian countries. HRD can maintain pancreatic β-cell identity by acting on the Notch1/Ngn3 pathway (Yuan et al., 2019). Meanwhile, HRD can treat T2DM by inhibiting the expressions of PEPCK and G6Pase and acting on the PI3K/Akt/FoxO1 signaling pathway. Meanwhile, it can increase the glycolysis-related gene and decrease the expression of the lipogenic gene (Wu et al., 2021). It has been further proven that the CR-GRER herb pair has a synergistic effect on compatibility (Wang et al., 2021). In addition, the combination of these two herbs may have clinical value in treating depression in diabetic patients (Zhang et al., 2021).

Based on the above analysis, we found that a combination of CR and GRER in a 1:1 ratio had a therapeutic effect on T2DM. At present, the clinical application of the CR-Renshen herb pair is limited, and there are still many directions to be explored in the study of this herb pair. Firstly, further clinical studies need to be enhanced to better evaluate its efficacy and safety. Secondly, it is also important to further study the mechanism of action and identify the active components of the CR-GRER herb pair, as well as their effects on the development of diseases. In addition, when combined with modern scientific and technological methods, such as genomics and proteomics, we can deeply understand the molecular mechanisms and targets of the CR-GRER herb pair.

### 3.6 *Coptidis rhizoma* and *Aucklandiae radix*

AR is pungent and bitter in flavor and warm in nature, and has the functions of promoting qi circulation to relieve pain and invigorating the spleen to promote digestion. It is often used for the treatment of asthma, influenza, toothache, leprosy, coughs, and rheumatism (Seo et al., 2015). Costunolide and dehydrostuslactone are the primary components found in AR (Zhang et al., 2014). The CR-AR herb pair originated from the famous monograph *Prescriptions of the Pharmacy Bureau* (和剂局方). This herb pair is known for its ability to clear heat and dry dampness, promote Qi circulation, relieve pain, and is used for treating enteritis, bacillary dysentery, and alleviating pain. XLP is composed of AR prepared with CR (Yu EF) and AR at a ratio of 4:1, which were made into pills with vinegar (Chinese Pharmacopoeia Commission, 2020). XLP is a famous ancient formula for treating gastrointestinal diseases, such as diarrhea, GC and UC, and is commonly used in clinical practice nowadays. A study showed that XLP can alleviate UC by suppressing inflammation and blocking the activation of the PI3K/Akt/mTOR pathway (Wang et al., 2021). The combination of CR and AR can attenuate UC by regulating the TLR4/MyD88/NF-κB pathway (Dai et al., 2023). Additionally, XLP can improve antibiotic-associated diarrhea (AAD) by reducing mucosal damage and increasing the expression of claudin-1 and occludin (Yang et al., 2021). A study has indicated that dehydrocostus lactone and berberrubine are the two main active components in XLP with anti-GC effects (Yu et al., 2023). Wan et al. (2008) investigated the berberine content in different proportions (1:1, 2:1, 3:1, 4:1, 5:1, 6:1, 7:1, 8:1, and 9:1) of CR and AR in XLP. The experimental results suggested that the highest berberine content in CR was obtained with a ratio of 1:1 among the nine different ratios. This indicates that increasing the proportion of AR was beneficial for enhancing the dissolution of active ingredients in CR. However, the results of pharmacological and bacteriostatic experiments showed that the best anti-inflammatory and analgesic effects, as well as the lowest inhibitory concentration, were observed when the ratio of CR and AR was 5:1. It is confirmed that the efficacy of Chinese herbal medicine does not depend solely on the number of active ingredients, but rather on the pharmacodynamic effect of the ratio between them. Therefore, the optimal ratio of CR and AR in XLP was determined to be 5:1. To further investigate variations of bioactive components after combining the two herbs, Wan et al. (2009) also studied the pharmacokinetics of alkaloids in the extracts of the CR-AR herb pair in the plasma of rats. The results demonstrated that the peak time of berberine in the CR-AR herb pair section was faster than that in the CR extract alone, and its maximum plasma concentration was higher than that in the extract. This indicates that AR can improve the dissolution of berberine in CR.

According to the above analysis, the synergistic effects of the CR-AR herb pair vary with different compatibility ratios. The current research on the CR-AR herb pair mainly focuses on the chemical composition, pharmacological effects, and clinical applications. However, we believe that there are still many directions to be explored in the study of the CR-AR herb pair. Firstly, the active ingredients and their mechanisms of action can be studied in depth to reveal their specific modes of action in the treatment of diseases. Secondly, the active and inactive components in plasma can be further evaluated through an in-depth study of the CR-AR herb pair using medicinal chemistry, pharmacodynamics, and other technologies. From there, the active components can be extracted and purified, and their properties, such as pharmacodynamic evaluation and pharmacokinetics can be studied. To provide a foundation for its better clinical application in Chinese medicine.

## 4 Discussion

Herb compatibility is the core of TCM prescription and is closely related to treatment effectiveness. We reviewed the bioactive components and compatibility effects of CR and its herb pairs and discovered that the bioactive components may change *in vivo* and *in vitro*. Complex physical and chemical reactions may accompany the decocting process of herb pairs, and the interaction of these chemical substances could lead to changes in the bioactive ingredients, thus adapting to complex conditions to increase effectiveness, reduce toxicity, broaden the scope of treatment, or prevent herb poisoning. Therefore, it is extremely important to study the changes in bioactive components and the compatibility effect. In addition, after the herb pairs enter the body, various chemicals can either promote or inhibit ADME (absorption, distribution, metabolism, and excretion) processes. The pharmacokinetic study is an essential component in elucidating the mechanism of action of herbs. These findings provide a foundation for future research to understand the influence of herb pairs and their applications in clinical settings.

In recent years, some progress has been made in the study of the compatibility effects of certain herb pairs containing CR, which lacks a certain degree of systematicity. Taken together, Table 2 summarizes the relevant research of the compatibility effects of CR and other major herbs. In pharmacology, an increasing number of studies are investigating the underlying mechanisms of herb pairs by observing physiological and pathological indicators and conducting multi-omics analysis (Zhang et al., 2024). However, there is also room for improvement in certain aspects. In terms of material basis, most scholars primarily conduct qualitative and quantitative studies focusing on individual major active ingredients (Zhang et al., 2024). The analysis has failed to comprehensively examine the differences in all chemical components before and after the compatibility of herb pairs. Plant metabolomics technology utilizes a variety of testing techniques to conduct comprehensive qualitative and quantitative analyses to determine the pattern of changes in the metabolism of components. At present, it has been widely used in the identification of Chinese herbal medicine varieties, planting and cultivation, assessing the impact of external factors on the quality of roots and rhizomes, as well as in harvesting and processing (Zhao et al., 2012; Díaz et al., 2014; Y. Yang et al., 2023). Therefore, the concept of plant metabolomics can also be utilized to examine the chemical composition of herb pairs after compatibility. The variations in chemical composition before and after compatibility can be analyzed comprehensively. In addition, supramolecular research has also been applied in the study of chemical components, such as CR-GRR (Zhu et al., 2018). It presents a novel approach to uncovering the pharmacodynamic material basis of traditional herbs and investigating the bioactive ingredients and pharmacological effects of herb pairs. In terms of compatibility effects, analyzing a single or a few active ingredients may overlook the interactions among multiple components. Therefore, bridging the gap between the chemical composition analysis of herbal pairs and pharmacokinetic mechanism studies involves integrating the pharmacokinetic research techniques of traditional Chinese medicine multi-components into the study of pharmacokinetic mechanisms after the compatibility of herbal pairs (Li et al., 2018). Additionally, the Spectral-efficacy relationship of traditional herbs is a new idea for the study of the material basis of the efficacy of Chinese medicine. The combination of “spectrum” and “effect” to explain the interrelationship between the chemical constituents and the efficacy, to elucidate the material basis of the efficacy of traditional herbs. The composition of Chinese herbal medicine is complex, and the metabolic efficiency and pathways of its various components vary among individuals. Strengthening the research on potential changes in chemical composition and the emergence of new compounds of CR and its herb pairs before and after compatibility is crucial. Additionally, integrating metabolomics and other technologies to further explore the pharmacological effects and mechanisms of CR and its combinations after ingestion can enhance clinical applications. This lays a foundation for the in-depth study and development of herb pairs containing CR.

**TABLE 2:** Studies on pharmacological effects of the combination of *Coptidis rhizoma* and other herbs.

Herb pairs	Bioactive constituents	Ratio	Efficacy	Diseases	Animal/model (vivo/vitro)	Dose/Concentration	Positive controls	Duration	Targets	Refs
CR and EF	Water extract of ZJP	6:1	Improving the GES-1 damage	Chronic atrophic gastritis (CAG)	Rats: MNNG 170 μg/mL (free drinking) and 1 mL/100g (ig) Tong et al. (2021a) and Tong et al. (2021b), GES-1 cells: MNNG 10, 20, 40, 60, 80, 100 μМ Tong et al. (2021a)	Rats: 0.63, 1.26, 2.52 g/kg Tong et al. (2021a) and Tong et al. (2021b), GES-1 cells: 10, 15, 30, 40, 60, 80, 100, 200, 400, 600, 800, 1,000 μg/mL Tong et al. (2021a)	Rats: Vitamin tables: 200 mg/kg	Rats: 28 d Tong et al. (2021a) and Tong et al. (2021b), GES-1 cells: once Tong et al. (2021a)	TGF-β1/PI3K/Akt↓	Tong et al. (2021a) and Tong et al. (2021b)
	Water extract of ZJP	6:1	Reducing inflammation	CAG	Rats: *Helicobacter pylori (H. pylori)* 1.5 × 108 CFU/mL (ig), GES-1 cells: *H. pylori* (at multiplicities of infection of 10:1, 20:1, 50:1 and 100:1)	Rats: 0.63, 1.26, 2.52 g/kg, GES-1 cells: 0, 10, 20, 30, 40, 60, 80, 120 and 160 μg/mL	Rats: Omeprazole: 1.8 mg/kg	Rats: 28 d, GES-1 cells: 0, 6, 12, 24 h	JMJD2B/COX-2/VEGF↓, HMGB1↓, MyD88↓, RAGE↓, NF-κB p65, RAS↓	Wen et al. (2022)
	Water extract of ZJP	6:1	Anti-ulcerogenic	Gastric ulceration	Mice: 0.2 mL/kg ethanol	Mice: 1, 2 g/kg	Omeprazole 20 mg/kg	Mice: once	NF-кB↓, IL-6/TNF-α↓	J.Wang et al. (2015)
	Ethanol extract of ZJP	6:1	Inhibiting the expression of inflammatory mediators	Gastric ulcer, gastroesophageal reflux disease, gastritis	RAW 264.7 cells: lipopolysaccharide 100 ng/mL	RAW 264.7 cells: 0.1, 1, 10, 100 μg/mL	—	RAW 264.7 cells: once	iNOS↓, COX-2↓, IL-6↓, IL-1β↓, TNF-α↓	Wang et al. (2012)
	Water extract of ZJP	6:1	Ameliorating inflammation	Gastric injury	Rats: indomethacin 5 mg/kg	Rats: 1.26, 2.52, 5.04 g/kg	Omeprazole: 1.8 mg/kg	Rats: 7 d	p-ERK/ERK↓, p-P38/P38↓, p-JNK/JNK↓	Wei et al. (2021a)
	ZJP	6:1	Decreasing the expression of proinflammatory cytokines	Colitis	Mice: 3% (w/v) DSS (free drinking)	Mice: 3 g/kg	5-ASA 300 mg/kg	Mice: 7 d	PI3K↓, p-Akt↓, TSC1↑, TSC2↑, Rheb↓, Raptor↓, Rictor↑	Zhou et al. (2020)
	Water extract of ZJP	6:1	Reshaping gut microbiota and modifying metabolites	UC	Rats: 4% (w/v) DSS (free drinking)	Rats: 2.8 g/kg	—	Rats: 6 d	—	Cai et al. (2022)
	Water extract of ZJP and Fan-ZJP	6:1; 1:6	Alleviating inflammation	UC	Mice: 5% (w/v) DSS (free drinking)	Mice: 2.3, 4.6 g/kg	Salicylazosulfapyridine: 20 mL/kg	Mice: 7 d	TNF-α↓, IL-10↑	Luo et al. (2010)
	ZJP	6:1	Anti-tumor	Pancreatic cancer	BxPC-3 and Panc-1 cell	BxPC-3 and Panc-1 cell: 0, 100, 200, 300, 400, 500, 600, 700, 800, 900 μg/mL	—	BxPC-3 and Panc-1 cell: once	EGFR↓, AKT↓, PI3K↓, BAD↑, Cleaved Caspase 9↑, Cleaved Caspase 3↑	K.Wang et al. (2021)
	ZJC	6:1	Anti-tumor	Colorectal cancer	Nude mice: injecting 1 × 107 HCT116 cells, HCT116, HT29 and SW480 cells	Nude mice: 78.75, 157.5, 315 mg/kg, HCT116, HT29 and SW480 cells: 0, 12.5, 25, 50, 100 and 200 μg/mL	Nude mice: Capecitabine 130 mg/kg	Nude mice: 26 d, HCT116, HT29 and SW480 cells: once	P53↑,CDKN1A↑, E2F1↓,CDK2↓, BCL2↓, PRKCB↓, MYC↓, MMP9↓	Fan et al. (2022)
	Ethanol extract of ZJP	6:1	Anti-tumor	Colorectal cancer	SW403 cells	SW403 cells: 25, 50, 100 μg/mL	—	SW403 cells: once	Wnt/β-catenin↓	Pan et al. (2017)
	Berberine and evodiamine	1:4, 1:2, 1:1, 2:1, 4:1	Anti-tumor	Colorectal cancer	Caco-2 and NCM469 cells	Caco-2 and NCM469 cells: berberine and evodiamine (2.5 and 10 μM, 5 and 10 μM, 20 and 10 μM, 20 and 10 μM, 40 and 10 μM	—	Caco-2 and NCM469 cells: once	P-gp↓	Guan et al. (2020)
	Quercetin	6:1	Anti-tumor	Colorectal cancer	HCT116 and HT29 cells	HCT116 and HT29 cells: 100, 150, 200, and 250 μΜ	—	HCT116 and HT29 cells: once	PI3K/AKT↓, Bacl2↓, Bax↑	S.Huang et al. (2020)
	Water extract of ZJP	6:1	Anti-tumor	Cancer	Mice: 2 × 10^5^ cells/mL S180 tumor cells (subcutaneous injection)	Mice: 42, 36 and 6.0 mg raw material/mL	Mice: Cyclophosphamide 20 mg/kg	Mice: 10 d	Bax↑, P53↑	Wang et al. (2009)
	Water extract of ZJP	6:1	Anti-tumor	Hepatocellular carcinoma	HepG2 cell line	HepG2: 1 mg/mL	—	HepG2: once	AP-1/NF-κB↓	Chao et al. (2011)
	Berberine chloride and evodiamine	—	Anti-tumor	Melanoma	B16 Melanoma cells	Melanoma B16 cells: berberine chloride and evodiamine: 18.6 and 2.25, 9.3 and 1.13, 4.65 and 0.56, 2.32 and 0.28, 1.16 and 0.14 μg/mL	—	—	—	Lin et al. (2020)
	ZJP	6:1	Anti-hypertension	hypertension	SHR Rats	Rats: 0.9, 3.6 g/kg	Nimodipine tables: 2.25 mg/kg	Rats: 28 d	—	Bai (2009)
	Water extract	3:1	Anti-hypertension	hypertension	SHR Rats	Rats: 10 mL/kg	Nifedipine tables: 1 mg/kg	Rats: 28 d	—	Chu et al. (2008)
	Water extract	1:1	Anti-hyperlipemia	Hyperlipidemia	Mice: high fat feed	Mice: 0.75, 1.5, 3/kg	Atorvastatin:1.5 mg/kg	Mice: 56 d	FXR↓, FGF15↓, FGFR4↓, CYP7A1↑, FXR↑	Xu (2020)
	ZJP, GLP, ZYP	6:1/2:1/1:1	Anti-hyperlipemia	Hyperlipidemia	Mice: 0.5 mL/pcs High fat emulsion, 0.1 mL/10 g 75% Egg yolk emulsion	Mice: 4 g/kg	Simvastatin: 0.04 g/kg	Mice: 10 d	—	Shen (2007)
	Berberine and evodiamine	1:1	Anti-hyperlipemia	Hyperlipidemia	Rats: high fat feed	Rats: 44.6, 89.2 mg/kg	Simvastatin: 7 mg/kg	Mice: 56 d	JAK2/STAT3↓	Zhou et al. (2018)
	Water extract of	6:1/2:1/1:1/1:6	Antipyretic	—	Rats: 10 mL/kg 15% yeast suspension	Rats: 0.20 g/mL	Aspirin Enteric-coated tablets: 2 mg/mL	Rats: once	—	Zhang et al. (2017)
	Ethanol extract	6:1/2:1/1:1/1:6	Antipyretic	—	Rats: heat exposure	Rats: 2.4 g/kg	Huoxiang Zhengqi Shui: 0.2 mL/100g	Rats: 3 d	—	Gao et., 2010
	GLP	2:1	Regulating TNF signal pathway	Nonalcoholic steatohepatitis	—	—	—	—	AKT1	R. Gao et al. (2022)
	Zoagumhwan	6:1	Anti-inflammation	Endothelial inflammation	HUVEs cells and U937 cells: Angiotensin II	HUVEs cells, U937 cells: 1, 10 μg/mL	—	—	MCP-1↓	Ko et al. (2007)
	Ethanol extract of ZJP	6:1	Anti-inflammation	—	Raw 264.7 cells: LPS 100 ng/kg	Raw 264.7 cells: 1, 10, 100 mg/L	Fluoxetine: 7.5 mg/kg	Mice: once	TNF-α↓, COX-2↓, iNOS↓, IL-6↓, IL-1β↓	Wang et al. (2012)
	Water extract of ZJP	6:1	Anti-depression	Under chronic unpredictable mild stress	Rats: CUMS procedures	Rats: 0.6, 1.2 g/kg	Fluoxetine (15 mg/kg)	Rats: 35 d	—	Wang et al. (2020)
	Ethanol extract of ZJP	6:1	Anti-depression	—	Mice: tail suspension test, forced swimming test and head impulse test	Mice: 5, 20 mg/kg (forced swimming test), 5, 10 mg/kg (forced swimming test), 5, 10, 20 mg/kg head impulse test)	Fluoxetine: 7.5 mg/kg (tail suspension test and forced swimming test)	Mice: once	—	Q.S. Wang et al. (2013)
	ZJP	6:1	Anti-depression	—	Mice: CUMS procedure (X. Xu et al., 2020), PC12 cells: 200 μM corticosterone	Mice: 225, 450, 910 mg/kg, PC12 cells: 225, 450, 910 mg/kg	Mice: Fluoxetine:10 mg/kg	Mice: 21 d	TPH2/5-HT↑	Y. Wang et al. (2023)
CR and SR	Baicalin and berberine hydrochloride	3:1/1:1/1:3	Maintaining the balance of proinflammation cytokines and anti-inflammation cytokines	Colitis	Raw264.7 cells: LPS 1 g/mL, Mice: dextran sulfate sodium	Raw264.7 cells: 5, 7.5, 10 μg/mL (3:1, 1:1 and 1:3), Mice: 20, 40, 80 mg/kg (1:1)	Mice: Sulfasalazine: 125 mg/kg	Mice: 7d	—	Yan et al. (2022)
	Water extract	1:1	Improving glucose and lipid metabolism	T2DM	Rats: high-fat diet along with 30 mg/kg streptozocin (STZ)	Rats: 6.3, 12.6 g/kg	Metformin: 0.09 g/kg	Mice: 1 month	MAPK↓, PI3K/Akt↑	Cui et al. (2018)
	Water extract	1:1	Ameliorating the gut microbiota composition and glycolipid metabolism	T2DM	Rats: high-fat diet and 30 mg/kg STZ	Rats: 6.3 g/kg	—	Rats: 1 month	—	Xiao et al. (2020)
	Water extract	1:1	Antihyperglycemic	Diabetic	Rats: 55 mg/kg STZ	Rats: berberine (100 mg/kg) + baicalin(125 mg/kg)	Acarbose: 20 mg/kg	Rats: 33 d	—	Liu et al. (2013)
	Water extract	1:1	Alleviating inflammation	UC	Mice: 3.5% (w/v) DSS (free drinking)	Mice: 9 g/kg	—	Mice: 7 d	—	Liu et al. (2022)
CR and MOC	Water extract	1:1	Anti-H1N1 influenza	pneumonia	Mice: intranasally infected with 10-fold LD50 influenza virus in 30 µL PBS	Mice:4, 8, 16 g/kg	Oseltamivir phosphate capsules 75 mg/kg	Mice: 5 d	TLR3/TLR7/MyD88/NF-κB p65↓	Zhang et al. (2021)
	Ethanol extract	1:1	Alleviating inflammation	UC	Mice: 3% (w/v) DSS (free drinking)	Mice: 160, 320 mg/kg	—	Mice: 7 d	—	Cheng et al. (2023)
	Water extract	3:2	Anti-inflammatory	UC	Mice: 3% (w/v) DSS (free drinking)	Mice: 1.25, 2.5, 5 g/kg	Sulfasalazine: 0.4 g/kg	Mice: 10 d	5-HT3A/NK-1R/VPAC1↓, VPAC1↑	Wang et al. (2022)
	Water extract	1:1	Anti-inflammatory	UC	Rats: 40 mg/kg 2,4,6-trinitrobenzene sulfonic acid	Rats: 1, 2, 4 g/kg	Sulfasalazine: 0.4 g/kg	Mice: 7 d	TGF-β1↑	Xie et al. (2022)
CR and GRR	Berberine + Glycyrrhizin	1:1	Attenuating ischemia–reperfusion injury	Stroke	Mice: tMCAO	Mice: 50 mg/kg	—	Mice: 14 d	HMGB1/NF-κB↓	Zhu et al. (2018)
	Water extract	3:2	Anti-diarrhea	Diarrhea	Mice: 0.3 g/mL senna	Mice: 15.21 mL/kg	—	Mice: once	—	Yu et al. (2005)
CR and GRER	Water extract	1:1	Inhibiting hepatic glucose production	T2DM	db/db mice, HepG2: glucagon	db/db mice: 0.3g, 0.6 g/mL	—	Mice: 28 d	PI3K/Akt/FoxO1↑	Wu et al. (2021)
	Berberine and ginsenoside Rb1 mixture	—	Improving the expression of neurotrophic factor and downregulating the levels of cortisol	Diabetes with depression	Rats: high-fat feed and 30 mg/kg STZ, and then ice water, 45°C, withdrawal of food and water, 45 °C thermal stimulation, noise exposure, tail-pinching, reversed dark/light cycle, cage tilting at 45°C, wetting of bedding materials	Rats: berberine (150 mg/kg) + ginsenoside Rb1 mixture (20 mg/kg)	Metformin (0.18 g/kg) + fluoxetine hydrochloride (1.8 mg/kg)	Rats: 28 d	Brain-derived neurotrophic factor↑	Zhang et al. (2021)
	Water extract	1:1	Improving glucose metabolism	T2DM	db/db mice	db/db mice: 3.03, 6.06 g/kg	Saxagliptin: 10 mg/kg	Rats: 56 d	Notch1↑, Ngn3↓	Yuan et al. (2019)
CR and AR	Water extract	4:1	Suppressing inflammation	UC	Mice: 3% (w/v) DSS (free drinking)	Mice: 1.35, 2.7, 5.4 mg/g	5-amino salicylic acid (5-ASA): 0.45 mg/g	Mice: 7d	TLR4/MyD88/NF-κB↓	Dai et al. (2023)
	Xianglian Pill	4:1	Suppressing inflammation	UC	Mice: 3% (w/v) DSS (free drinking)	Mice: 0.8, 1.6, 3.2 g/kg	5-ASA: 50 mg/kg	Mice: 7d	PI3K/Akt/mTOR↓, Beclin-1↑, claudin-1↑ zonula occludens-1↑	Wang et al. (2021)
	Dehydrocostus lactone and berberrubine	—	Inhibiting cell cycle and promote cell apoptosis	gastric cancer	GES-1, MGC-803, AGS, HGC-27, SGC-7901 cells	GES-1, MGC-803, AGS, HGC-27, SGC-7901 cells: 0–100 μmol/L	—	—	Caspase3↑, Bcl/Bax↓	Yu et al. (2023)
	Ethanol extract	1:1, 2:1, 3:1, 4:1, 5:1, 6:1, 7:1, 8:1, 9:1	Relieving pain	Pain	Mice: 0.6% acetic acid	Mice: 27.5 mg/mL	Xiaoyan Zhiling Pian: 6.15 mg/mL	Mice: 7d	—	Wan et al. (2008)
	Ethanol extract	1:1, 2:1, 3:1, 4:1, 5:1, 6:1, 7:1, 8:1, 9:1	Anti-inflammatory	Inflammation	Mice: 0.05 mL xylene	Mice: 27.5 mg/mL	Xiaoyan Zhiling Pian: 6.15 mg/mL	Mice: 5d	—	Wan et al. (2008)
	Xianglian Pill	4:1	Attenuating the inflammatory disorders	AAD	Mice: cefuroxime and levofoxacin (300 mg/kg + 200 mg/kg)	Mice: Xianglian Pill (500, 1000, 2000 mg/kg/d) + cefuroxime and levofloxacin (300 mg/kg/2d + 200 mg/kg/2d)	—	Mice: 5d	claudin-1/occludin↑	Yang et al. (2021)

Modern scholars have extensively researched numerous renowned herb pairs for their compatibility effects, such as CR-SR, and CR-EF. It may be related to their origins in the famous classical ancient formulas, HLJDD and ZJP. Through the preliminary data compilation, we found that certain combinations, such as CR and ASR, CR and *PCC*, and CR and *Saposhnikoviae radix* herb pairs are fewer studies, although they have high frequency. Combined with modern evaluation techniques and innovative research concepts, these herb pairs with a higher frequency of usage are more deserving of in-depth study regarding pharmacological effects and variations of bioactive ingredients after the combination of herbs. These data can also provide valuable references for researchers, offering guidance for research directions and clinical practices, as well as facilitating drug development.

## 5 Conclusion

Studying herb pairs and conducting numerous experiments and clinical observations can provide insights into the development and utilization of new drugs, as well as their effective application in the clinical practice of TCM. These findings provide a foundation for future research to understand the influence of herb pairs and their applications in a clinic. Even if the combination of herbs is effective, it could take some time to identify the compatibility effects and the main active ingredients. In investigations of multi-herb formulations, the efficacy-driven approach is now extensively used, with efficacy being the first aspect to be proven. Although the ultimate purpose of studying the compatibility of herb pairs is to provide reasonable, safe, and effective indications for practical clinical use and prescription, the investigation of traditional uses, bioactive components, and compatibility effects remains an inevitable topic for the further development of traditional Chinese formulations. In conclusion, the key traditional uses, bioactive components, and compatibility effects of CR and its herb pairs were systematically summarized and synthesized through literature retrieval. It was found that combining CR with other herbs often resulted in superior effects compared to using them individually, expanding the scope of therapeutic applications. We hope that this information can serve as a reference for researchers, facilitating experimental studies and the development of new drugs.

## Figures and Tables

**Table:** 

**5-HT**	5-hydroxytryptamine
**AAD**	antibiotic-associated diarrhea
**Akt**	protein kinase B
**AP-1**	activator protein 1
**BAD**	BCL2 associated agonist of cell death
**CDKN1A**	cyclin-dependent kinase inhibitor 1A
**COX-2**	cyclooxygenase-2
**CYP7A**	cholesterol 7alpha-hydroxylase
**EGFR**	epidermal growth factor receptor
**ERK**	extracellular regulated protein kinases
**FGF**	fibroblast growth factor
**FGFR**	fibroblast growth factor receptor
**FXR**	farnesoid X receptor
**GC**	gastric cancer
**GGQLD**	Gegen Qinlian Decoction
**GLP**	Ganlu Power
**H1N1**	influenza a
**HHD**	Huanglian Houpo Decoction
**HLJDD**	Huanglian Jiedu Decoction
**HMGB1**	high-mobility group box 1
**HRD**	Huanglian Renshen Decoction
**IL-6**	interleukin-6
**IL-1β**	interleukin-1β
**IL-10**	interleukin-10
**iNOS**	inducible nitric oxide synthase
**JAK2**	Janus kinase 2
**JNK**	c-Jun N-terminal kinase
**MAPK**	mitogen-activated protein kinase
**mTOR**	mammalian target of rapamycin
**MyD88**	Myeloid differentiation primary response protein 88
**NF-κB**	nuclear factor kappa-B
**P-gp**	p-glycoprotein
**PI3K**	phosphatidylinositol 3-kinase
**RAGE**	receptor for advanced glycation end-products
**STZ**	streptozocin
**T2DM**	type 2 diabetes mellitus
**TPH**	tryptophan hydroxylase
**STAT3**	signal transducer and activator of transcription 3
**TCM**	Traditional Chinese Medicine
**TG**	triglyceride
**TGF-β1**	transforming growth factor β
**TLR**	toll-like receptor
**TNF**	tumor necrosis factor
**TSC**	tuberous sclerosis complex
**UC**	ulcerative colitis
**VEGF**	vascular endothelial growth factor
**VPAC**	vasoactive intestinal peptide receptor type
**XLP**	Xianglian Pill
**ZJP**	Zuojin Pill
**ZYP**	Zhuyu Pill
