# Supplementary material for: Several major herb pairs containing Coptidis rhizoma: a review of key traditional uses, constituents and compatibility effects
Source: Front Pharmacol. 2024 Jun 25;15:1399460. doi: 10.3389/fphar.2024.1399460 (PMC11231094; doi:10.3389/fphar.2024.1399460)
Supplement: Supplementary file 1 [file Table1.docx]

**TABLE S1** The species of seven herbs in botany, phytochemistry, and pharmacology

| Latin name | Medicinal  parts | Botanical sources | Botany ^a^ | Chemical compounds | Pharmacological effects |
| --- | --- | --- | --- | --- | --- |
| *Coptidis rhizoma* | Rhizome | *Coptis chinensis* Franch. | Yellow branched rhizomes. The leaves are slightly leathery, ovate-triangular, with three lobes. During the fruiting period, there are one or two peduncles, measuring 12-25 cm long. This plant has 3-8 flowers clustered into dichotomous or polytomous umbels. The bracts are lanceolate. The sepals are yellow-green and elongated oval-shaped, measuring 9-12.5 mm in length and 2-3 mm in width. The petals are linear or lanceolate, with approximately 20 stamens. There are 8-12 carpels, each with slightly outwardly curved styles. There are 7-8 elliptical seeds, approximately 2mm long and 8mm wide, brown in color. Flowering occurs from February to March, and the fruit is commonly harvested from April to June. Normally growing in mountainous forests or shady valleys at altitudes ranging from 500 to 2000 meters, both in the wild and cultivated. | Alkaloids, organic acids, coumarins, phenylpropanoids, quinones, and other chemical components (Wang, Wang, et al., 2019). | Anti-pathogenic microorganism activity, Protective effects on the cardiovascular system, Protective effect against ischaemic heart disease, Antidiabetes, Anticancer, and other effects (Wang, Wang, et al., 2019). |
|  |  | *Coptis deltoidea* C. Y. Cheng et Hsiao. | Yellow rhizomes with few or no branches. The leaves are slightly leathery, elliptical, and have three lobes. During the fruiting period, there are one or two flower stems slightly longer than the leaves. Additionally, 4-8 flowers cluster into a polytomous umbel inflorescence. The bracts are linear and lanceolate. The narrow ovate sepals are yellow-green, measuring 8-12.5 mm in length and 2-2.5 mm in width. There are approximately 10 petals. About 20 stamens are approximately half the length of the petals. The anthers are yellow, and the filaments are narrowly linear. There are 9-12 carpels with slightly curved styles. The follicles are 6-7 millimeters long. Flowering occurs from March to April, and the fruit is commonly harvested from April to June. It usually grows in mountain forests at altitudes of 1600-2200 m. |  |  |
|  |  | *Coptis teeta* Wall. | The rhizomes are yellow, densely noded, with numerous adventitious roots. The leaves are ovate-triangular, measuring 6-12 cm in length and 5-9 cm in width, deeply lobed into three parts. During the fruiting period, there are one or two flower stems reaching a height of 15-25 cm. This plant has 3-5 flowers clustered into a compound umbel inflorescence. The bracts are elliptical. The yellow-green elliptical sepals measure 7.5-8 mm in length and 2.5-3 mm in width. The anthers are about 0.8 mm long, and the filaments are 2-2.5 mm long. There are 11-14 carpels with outwardly curved styles. The follicles are 7-9 mm long and 3-4 mm wide. It typically grows in cool and humid mountainous areas at altitudes of 1500-2300 meters in shady locations. |  |  |
| *Euodiae* *fructus* | Fruit | *Euodia rutaecarpa* (Juss.) Benth. | This is a small tree or shrub, typically reaching a height of 3-5 m, with young branches being dark purple-red. It has 5-11 leaflets that are thin, papery, and ovate, elliptic, or lanceolate in shape. The flowers are borne in terminal inflorescences. The male flowers are spaced apart from each other, while the female flowers are either densely clustered or spaced apart. Both types of flowers have 5 sepals and petals, occasionally 4, arranged in a clasping manner. The petals of male flowers are 3-4 mm long, with 4-5 deeply divided stamens. The petals of female flowers are 4-5 mm long, with reduced staminodes that are either scale-like or short-linear. Sometimes, they are accompanied by small sterile anthers. The fruiting structure is 3-12 cm wide, with fruits densely or sparsely arranged, dark purple-red, and with large oil spots. Each fruit segment contains one seed, which is nearly spherical, 4-5 mm long, glossy, and dark brown-black. Flowering occurs from April to June, and the fruit is commonly harvested from August to November. It grows in open forests or shrubby thickets on flat land up to elevations of 1500 meters, primarily on sunny slopes. | Alkaloids, terpenoids, flavonoids, phenolic acids, steroids, phenylpropanoids, anthraquinones, and essential oils (Li and Wang., 2020). | Anti-tumor, anti-inflammatory, anti-bacterial, anti-obesity, antioxidant, insecticidal effect, regulating central nervous system homeostasis, and cardiovascular protection (Li and Wang., 2020). |
|  |  | *Euodia rutaecarpa* (Juss.) Benth. var. *officinalis* (Dode) Huang. | The leaflets are papery. There are fewer fruits in the fruiting structure, which can be densely packed or more loosely arranged. It grows in low-altitude areas and is commonly cultivated in regions such as Zhejiang, Jiangsu, and Jiangxi. |  |  |
|  |  | *Euodia rutaecarpa* (Juss.) Benth. var. *bodinieri* (Dode) Huang. | The leaflets are thin and papery. The flowers on the female inflorescences are spaced apart, with petals approximately 4 mm long, and the fruit stalks are slender and elongated. It grows on mountain slopes in grassy areas or at the edges of forests. |  |  |
| *Scutellariae* *radix* | Root | *Scutellaria baicalensis* Georgi. | Perennial herbaceous plant. The rhizomes are thick and fleshy. Leaves are coriaceous, lanceolate to linear-lanceolate, 1.5-4.5 cm long, and (0.3) 0.5-1.2 cm wide. Inflorescences are terminal on stems and branches, often forming a conical cluster at the stem apex. Flower stalks are 3 mm long. Bracts are ovate-lanceolate to lanceolate. Sepals are 4 mm long when flowering, with shield-shaped appendages measuring 1.5 mm in height. Corollas are 2.3-3 cm long. There are 4 stamens, with flat filaments and slender styles. The disk is annular and 0.75 mm in height. The ovaries are brown and glabrous. Nutlets are ovoid, 1.5 mm high, 1 mm in diameter, blackish-brown, with tubercles, and a scar near the base on the ventral side. Flowering occurs from July to August, and fruiting takes place from August to September. Typically found on sunny, grassy slopes at elevations of 60-1300 (1700-2000) m. | Flavonoids, glycosides, and others (Wang et al., 2018). | Antioxidant, anti-inflammatory, liver protection, neuro-protection, anti-tumor, anti-bacterial, and anti-viral effects (Zhao et al., 2019). |
| *Magnoliae Officinalis cortex* | Bark | *Magnolia officinalis* Rehd.et Wils. | Trees, up to 20 m tall. The bark is thick and brown. Leaves are large and almost leathery. The flowers are white and fragrant. With approximately 72 stamens, 2-3 cm long, anthers 1.2-1.5 cm long, inwardly dehiscent, and filaments 4-12 mm long. Pistils are elliptic-ovate. Aggregate fruit is oblong-ovate. Samara has a beak that is 3-4 mm long. The seeds are triangular-ovoid, approximately 1 cm long. Flowering occurs from May to June, and fruiting takes place from August to October. This species is typically found in mountainous forests at elevations ranging from 300 to 1500 meters. | Lignans, alkaloids, volatile oils, and other compounds (Luo et al., 2019). | Antibacterial, anti-tumor, analgesic, anti-inflammatory, and anti-oxidative effects (Luo et al., 2019). |
|  |  | *Magnolia officinalis* Rehd. et Wils. var. *biloba* Rehd. et Wils. | The differences between this species and Magnolia officinalis lie in the following aspects: the leaves of this species have a slightly concave apex, forming two obtuse rounded shallow lobes, whereas the leaves of young seedlings have a bluntly rounded apex without being concave. The base of the aggregate fruit is narrower. Flowering occurs from April to May, and the fruit is in October. This plant species is typically found in forests at elevations ranging from 300 to 1400 meters. It is frequently grown near foothills and villages. |  |  |
| *Glycyrrhizae radix et rhizoma* | Root and rhizome | *Glycyrrhiza uralensis* Fisch*.* | Perennial herbaceous plants with thick roots and rhizomes. Stems are erect and branched. Leaves are 5-20 cm long. Axillary racemes. The bracts are lanceolate-ovate, brown, and membranous. The calyx is campanulate, with five lobes nearly equal in length to the calyx tube. Corolla is purple, white, or yellow. The ovary is densely covered with glandular hairs. Pods are curved, sickle-shaped, or ring-shaped, densely clustered into a sphere, with numerous tubercular projections and glandular hairs. It contains 3-11 dark green, round, or kidney-shaped seeds. Flowering occurs from June to August, and fruiting occurs from July to October. This plant is commonly found in dry sandy areas, riverbank sandy soils, mountain grasslands, and saline soils. | Triterpene saponins, flavonoids, glycyrrhiza polysaccharides, and other components (Chen et al., 2020). | Anti-inflammatory, anti-tumor, antibacterial, antiviral, and anti-oxidative effects (Chen et al., 2020). |
|  |  | *Glycyrrhiza inflata* Bat*.* | Perennial herbaceous plants with stout roots and rhizomes. Stems are erect and branched. Leaves are 4-20 cm long. Axillary racemes with numerous scattered flowers. Bracts are lanceolate-ovate. The calyx is campanulate with five teeth, which are equal in length to the calyx tube. Corolla is purple or light purple. Pods are ellipsoid or elongated, 8-30 mm long and 5-10 mm wide, straight or slightly curved. Seeds are 1-4, round, green, with a diameter of 2-3 mm. Flowering occurs from May to July, and fruiting occurs from June to October. Commonly found on river terraces, water edges, field edges, or wastelands. |  |  |
|  |  | *Glycyrrhiza glabra* L*.* | Perennial herbaceous plants have stout roots and rhizome stems that are erect and highly branched. Leaves are 5-14 cm long. Axillary racemes bear numerous densely packed flowers. The bracts are lanceolate and membranous, approximately 2 mm long. The calyx is bell-shaped, 5-7 mm long, with 5 lanceolate teeth that are nearly equal in length to the calyx tube. Corolla is purple or light purple. Pods are elongated and flattened, measuring 1.7-3.5 cm in length and 4.5-7 mm in width. They are slightly curved, resembling a sickle. They are 2-8 seeds, dark green, smooth, kidney-shaped, with a diameter of about 2 mm. Flowering occurs from May to June, and fruiting occurs from July to September. It is often found on river terraces, ditch edges, field edges, and roadsides, it can also thrive in relatively dry saline soils. |  |  |
| *Ginseng radix et rhizoma* | Root and rhizome | *Panax ginseng* C. A. Mey. | Perennial herbaceous plants with short rhizomes. The main root is enlarged, spindle-shaped, or cylindrical. Above-ground stems are solitary, reaching 30-60 cm in height. Leaves are palmately compound. An umbel inflorescence, approximately 1.5 cm in diameter, bears 30-50 flowers, rarely 5-6. The peduncles are usually longer than the leaves. There are five petals, five stamens, and two free carpels. Fruit is a flattened, reddish, ball-shaped structure. Seeds are kidney-shaped and milky white. This species is found in deciduous broad-leaved forests or mixed coniferous and broad-leaved forests at elevations of several hundred meters. | Ginsenoside, polysaccharide, volatile oil, protein, amino acids, organic acids, flavonoids, vitamins, and trace elements (S. Yang et al., 2023). | Exciting nerve center, anti -tumor, cerebrovascular protection, increasing immunity, delay aging, lowering blood lipid and fatigue (S. Yang et al., 2023). |
| *Aucklandiae radix* | Root | *Aucklandia lappa* Decne. | — | Terpenoids, glycosides, phenylpropanoids, steroids, and other compounds (Zhuang et al., 2021). | Anti-inflammatory, anti-cancer, Anti-gastric ulcers, Antibacterial, antifungal and other effects (Zhuang et al., 2021). |

^a^ Cited from the website: “https://[www.iplant.cn/foc](http://www.iplant.cn/foc).” “—” It is not recorded on the website “https://[www.iplant.cn/foc](http://www.iplant.cn/foc).”
