# Supplementary material for: Several major herb pairs containing Coptidis rhizoma: a review of key traditional uses, constituents and compatibility effects
Source: Front Pharmacol. 2024 Jun 25;15:1399460. doi: 10.3389/fphar.2024.1399460 (PMC11231094; doi:10.3389/fphar.2024.1399460)
Supplement: Supplementary file 2 [file Table2.docx]

**TABLE S2** Chemical structure of the main active constituents of the alkaloids in *Coptidis rhizoma*

| NO. | Compound | Structure | Molecular Formula | Refs |
| --- | --- | --- | --- | --- |
| 1 | Berberrubine |  | C_19_H_16_ClNO_4_ | Tripathi et al. (2007) |
| 2 | Berberine |  | C_20_H_18_NO_4_^+^ | M.T. Zhou et al. (2021) |
| 3 | berberamine |  | C_37_H_40_N_2_O_6_ | Gai et al. (2018) |
| 4 | Coptisine |  | C_19_H_14_NO_4_ | Lu et al. (2023) |
| 5 | Jatrorrhizine |  | C_20_H_20_NO_4_^+^ | Gai et al. (2018) |
| 6 | Epiberberine |  | C_20_H_18_NO_4_^+^ | Gai et al. (2018) |
| 7 | 1,3-Dioxolo[4,5-g]isoquinolin-5(6H)-one |  | C_10_H_7_NO_3_ | Wang, Wang, et al. (2019) |
| 8 | Choline |  | C_5_H_14_NO | Chen et al. (2012) |
| 9 | Columbamine |  | C_20_H_20_NO_4_ | Gai et al. (2018) |
| 10 | Groenlandicine |  | C_19_H_16_NO_4_^+^ | Gai et al. (2018) |
| 11 | Palmatine |  | C_21_H_22_NO_4_^+^ | Gai et al. (2018) |
| 12 | 8-Oxyberberine |  | C_20_H_17_NO_5_ | Wang et al. (2014) |
| 13 | 8-Oxycoptisine |  | C_19_H_13_NO_5_ | Gai et al. (2018) |
| 14 | Sanguinarine |  | C_20_H_14_NO_4_ | Wang, Wang, et al. (2019) |
| 15 | Oxysanguinarine |  | C_20_H_13_NO_5_ | Wang, Wang, et al. (2019) |
| 16 | Norsanguinarine |  | C_19_H_11_NO_4_ | Gai et al. (2018) |
| 17 | 6-Acetonyldihydrosanguinarine |  | C_23_H_19_NO_5_ | Wang, Wang, et al. (2019) |
| 18 | Tetrahydroberberine |  | C_20_H_21_NO_4_ | Wang et al*.* (2014) |
| 19 | Tetrandrine |  | C_38_H_42_N_2_O_6_ | Wang, Wang, et al. (2019) |
| 20 | Magnoflorine |  | C_20_H_24_NO_4_^+^ | T. Xu et al*.* (2020) |
| 21 | Berbithine |  | C_19_H_17_NO_5_ | Wang, Wang, et al. (2019) |
| 22 | 3-hydroxy-2-methoxy-9,10-methylenedioxy-8-oxo protoberberine. |  | C_19_H_15_NO_5_ | Wang, Wang, et al. (2019) |
| 23 | 6-([1,3]dioxolo[4,5-g]isoquinoline-5-carbonyl)-2,3-dimethoxy benzoic acid methyl ester |  | C_21_H_17_NO_7_ | Wang et al*.* (2014) |
| 24 | Noroxyhydrastinine |  | C_10_H_9_NO_3_ | Wang, Wang, et al. (2019) |
| 25 | Corydaldine |  | C_11_H_13_NO_3_ | Tource et al. (2016) |
| 26 | Thalifoline |  | C_11_H_13_NO_3_ | Gai et al. (2018) |
| 27 | Worenine |  | C_20_H_16_NO_4_ | Gai et al. (2018) |
| 28 | Chilenine |  | C_20_H_17_NO_7_ | Yang et al. (2014) |
| 29 | Corydine |  | C_20_H_23_NO_4_ | Gai et al. (2018) |
| 30 | 8-oxoepiberberine |  | C20H17NO5 | Gai et al. (2018) |
| 31 | Coptisonine |  | C_19_H_15_NO_6_ | Yang et al. (2014) |
| 32 | 8-Oxyberberrubine |  | C_19_H_15_NO_5_ | Wang, Wang, et al. (2019) |
| 33 | 8,13-Dioxocoptisine hydroxide |  | C_19_H_13_NO_7_ | Yang et al. (2014) |
| 34 | Berberastine |  | C_21_H_19_NO_5_ | Wang, Wang, et al. (2019) |
| 35 | Obamegine |  | C_34_H_34_N_2_O_6_ | Wang, Wang, et al. (2019) |
| 36 | 3-Hydroxy-1-(4-hydroxyphenethyl) pyrrolidine-2,5-dione |  | C_12_H_12_NO_4_ | Wang, Wang, et al. (2019) |
| 37 | Ehyl-2-pyrrolidinone-5(S)-carboxylate |  | C_7_H_11_NO_3_ | Wang, Wang, et al. (2019) |
| 38 | Methyl-5-hydroxy-2-pyridinecarboxylate |  | C_9_H_7_NO | Wang, Wang, et al. (2019) |
| 39 | 4-[Formyl-5-(hydroxymethyl)-1H-pyrrol-1-yl] butanoate |  | C_11_H_15_NO_4_ | Wang, Wang, et al. (2019) |
| 40 | 8,9-Dihydroxy-1,5,6,10-b-tetrahydro-2H-pyrrolo[2,1-a]-isoquinolin-5-one |  | C_12_H_13_NO_3_ | Wang, Wang, et al. (2019) |
| 41 | 1H-indole-3-carboxaldehyde |  | C_7_H_7_NO_3_ | Wang, Wang, et al. (2019) |
